# Toenail metal concentrations and kidney health among young people at risk of chronic kidney disease of uncertain etiology

**DOI:** 10.1097/EE9.0000000000000461

**Published:** 2026-02-09

**Authors:** Samantha M. Hall, Birgit Claus Henn, Selene Vences Brown, Juan José Amador Velázquez, Damaris López Pilarte, Magaly Rosario Amador Sánchez, Juan José Amador Sánchez, Yirong Yuan, Jocelyn Fimbres, Kathryn M. Rodgers, Brian P. Jackson, Maria Argos, Luis Carvalho, Madeleine K. Scammell, David J. Friedman, Daniel R. Brooks, Jessica H. Leibler

**Affiliations:** a Department of Environmental Health, Boston University School of Public Health, Boston, Massachusetts; b Centro Médico del Pacífico (CENMED), Masaya, Nicaragua; c Department of Earth Sciences, Dartmouth College, Hanover, New Hampshire; d Department of Mathematics and Statistics, Boston University, Boston, Massachusetts; e Division of Nephrology, Department of Medicine, Beth Israel Deaconess Medical Center and Harvard Medical School, Boston, Massachusetts; f Department of Epidemiology, Boston University School of Public Health, Boston, Massachusetts

**Keywords:** Chronic kidney disease of uncertain etiology, CKDu, Metals, Mixtures, Toenails

## Abstract

**Background:**

Metal exposure is a hypothesized risk factor for chronic kidney disease of uncertain etiology, an ongoing epidemic in Central America, yet metal biomonitoring data in this region are scant.

**Methods:**

We measured metal toenail concentrations using inductively coupled plasma mass spectrometry and calculated estimated glomerular filtration rate (eGFR) in a cohort of Nicaraguans 14–31 years (n = 297; 49% female; data collected in 2023). We used multivariable linear and ordinal regression and Bayesian kernel machine regression to examine individual element and mixture associations of arsenic, cadmium, mercury, nickel, lead, and uranium with eGFR.

**Results:**

The middle tertile of nickel was associated with −6.92 ml/min/1.73 m^2^ reduction in eGFR (95% confidence intervals [CI] = −10.7, −3.12) compared to the lowest tertile, suggesting a “*U*-shaped” association. The highest tertile of arsenic was associated with −4.25 ml/min/1.73 m^2^ eGFR reduction (95% CI = −8.42, −0.07) compared to the lowest tertile. Associations between other metals and eGFR were not detected. We observed no evidence of higher-order interactions or joint effects of the metal mixture on eGFR.

**Conclusions:**

In this sample of young people in a high-chronic kidney disease of uncertain etiology-risk region, nickel and arsenic were independently associated with reduced eGFR, but other metals and their mixture were not. This finding supports targeted metals biomonitoring and source investigation.

**What this study adds**

Our findings indicate highly elevated nickel concentrations in our sample compared to cohorts around the world. We observed consistency across regression models, with both arsenic and nickel associated with reduced kidney function. To our knowledge, our findings document among the strongest associations observed to date between metals and chronic kidney disease of uncertain etiology-related kidney outcomes in Central America, specifically among youth in a chronic kidney disease of uncertain etiology context. Notably, our findings are strengthened by the use of a long-term biomarker (toenails). We believe our Supplemental Discussion on biomarkers, reverse causation, and kidney health is of high relevance to the environmental epidemiology community and Journal readership.

## Introduction

Chronic kidney disease of uncertain etiology (CKDu) is an ongoing epidemic concentrated among rural and agricultural communities in tropical regions, including areas of Central America and South Asia.^1,2^ This disease has the highest documented prevalence among male agricultural laborers and generally presents in early adulthood, around 20–30 years.^3^ CKDu is a primarily tubular disease not associated with risk factors like diabetes or hypertension, unlike CKD in higher-income regions.^3^ The comprehensive cause of CKDu remains unclear; to date, research has been conducted almost exclusively among adults and has considered high heat stress, silica, metals, and agrochemical exposures in occupational settings, as well as a genetic vulnerability, in relation to disease risk.^4–7^

Early signals of kidney injury and dysfunction have been observed in children and adolescents living in regions of Central America, where the disease is termed Mesoamerican nephropathy (MeN),^8–11^ suggesting that early life environmental exposures and/or genetic factors may increase MeN risk. Young people are more susceptible than adults to harm from toxic exposures given ongoing growth and developmental processes.^12,13^ Nephrotoxic exposures and injuries during childhood and youth can impact long-term renal health trajectories.^14^ Understanding early disease processes and risk factors may provide important clues to the causes of the MeN epidemic.

Animal and human studies demonstrate nephrotoxic potential of some heavy metals and metalloids, including arsenic (As), cadmium (Cd), mercury (Hg), nickel (Ni), lead (Pb), and uranium (U).^15,16^ Exposure to some metals and metalloids (hereafter, “metals”) has been associated with elevated concentrations of kidney injury biomarkers,^17^ low estimated glomerular filtration rates (eGFR),^18^ increased risk of CKD,^19^ and increased organ metal accumulation in CKD patients.^20^ Mechanisms of kidney injury from metal exposure include increased oxidative stress in the nephron’s proximal tubule cells (PTs) through increased free radical and reactive oxygen species release (As, Ni),^15,21,22^ increased oxidative stress (As, Ni, and Pb),^15,22^ mitochondrial damage (As, Hg, and Pb),^16,23^ decreased PT transport and development (Hg, Pb),^15,24^ and increased cell death (Hg) from accumulation in the PTs (Cd).^25^ The nephrotoxic mechanisms of one metal may interact in nonadditive ways with coexposures, as demonstrated with other health endpoints;^26–28^ statistical methods accounting for exposure mixtures thus aid in the evaluation of health impacts.^29,30^

Elevated environmental concentrations of elements associated with industrial and agricultural activities, volcanic bedrock, and other geologic formations are widespread throughout Central America, specifically Nicaragua.^31–34^ Existing research on metals and MeN-relevant kidney function in Central America indicates mixed results, though exposure to some heavy metals (As, Cd, Pb) has been more strongly associated with CKDu risk in other hotspots, including Sri Lanka and India.^35,36^ Overall, little work in Central America has evaluated environmental exposures that may impact communities at risk of MeN outside of the workplace. In a small case-control study, Fischer et al^6^ identified a negative association between toenail Ni concentrations and acute kidney injury (AKI) in a sample of Nicaraguan sugarcane workers. Butler-Dawson et al^37^ found urine As and Cd to be negatively associated with eGFR among Guatemalan sugarcane workers. Levels of As in hair were positively associated with CKD risk in a case-control study of Mexican individuals by Bustamante-Montes et al.^38^ However, McClean et al. found no association between urine As, Cd, U, or blood Pb with eGFR in a group of Nicaraguan sugarcane workers.^39^ Smpokou et al^40^ also found no association between a panel of urine metals and eGFR status in a community cohort in Nicaragua. Notably, none of these studies estimated effects of metal mixtures, which reflect real-world conditions and may better describe nonadditive relationships between coexposures. Furthermore, while metal mixtures have been associated with non-CKDu-related kidney function among Mexican and American youth,^30,41,42^ no CKDu studies examining metals to-date have focused on youth, who are at elevated developmental risk from metal exposures.

To address these gaps, we used statistical methods for mixtures alongside traditional regression approaches to assess metal concentrations in toenails, a biomarker of longer-term metal exposure, in relation to eGFR within a population of youth at high risk of MeN. We hypothesized that higher concentrations of nephrotoxic metals would be associated with lower eGFR and that some metals interact to have greater than additive effects on eGFR.

## Methods

### Study design and cohort

This analysis utilized biological and questionnaire data collected in 2023 from participants of the Jovenes-Nica Study, a prospective cohort of male and female adolescents and young adults in Nicaragua (2022–2025), described previously.^8,11^ Briefly, youth were originally recruited in schools across four Nicaraguan regions with varying adult prevalences of CKD for evaluation of kidney health and environmental exposures. From 2022 to 2024, three rounds of annual follow-up were conducted by native Spanish-speaking, trained study personnel who administered detailed questionnaires covering health, occupation, diet, medical, and behavioral risk factors. Trained bioanalysts collected blood and urine specimens at each follow-up visit, which were maintained for <8 hours at 4 °C, then aliquoted and frozen at −80 °C for long-term storage. For this analysis, we used serum and toenails collected in 2023. For toenail metals analysis, we selected a sample of study participants from two regions with high CKD prevalence, Chinandega (municipality: Chichigalpa) and León (municipalities: Mina El Limón and La Paz Centro), to efficiently parallel the sex, age, and eGFR distribution of the study population in these regions (n = 297; 52.3% of the full cohort; N = 568). All parents and participants provided written informed consent, and protocols were approved by the Boston University Medical Campus Institutional Review Board and the Nicaraguan Ministry of Health.

### Exposure assessment

All toenails were cleaned of visible dirt with alcohol wipes before collection. Participants were instructed to cut toenails using sterilized stainless-steel clippers from all 10 toes during in-person study visits with guidance and assistance from study team members. Toenail clippings were collected into individually labeled paper envelopes, then stored and shipped at ambient temperature until analysis. Toenail specimens were processed using inductively coupled plasma mass spectrometry (IC-PMS) for a basic panel of 23 elements at the Dartmouth Trace Element Analysis Core (Supplemental Methods; https://links.lww.com/EE/A411). We used the median detection limits as the limits of detection (LOD). Full quality assurance and quality control procedures are summarized in Table S1; https://links.lww.com/EE/A411.^43^

### Outcome assessment

We calculated eGFR with the serum creatinine-based European Kidney Function Consortium equation with the *Q* value from Pottel et al used to age- and sex-normalize serum creatinine, an approach which has been validated in North American, African, and European populations.^44–46^ We modeled eGFR both as continuous and ordinal outcome variables with the following eGFR categories reflecting increasingly worse kidney function:^47^ >90 (referent, healthy function); 76–90; ≤75 ml/min/1.73 m^2^.

### Statistical analysis

Metal concentrations were natural log-transformed (ln) to improve normality of residuals and *z*-standardized for comparability. We selected six metals of primary interest to assess in main models (As, Cd, Hg, Ni, Pb, and U) based on (1) nephrotoxic potential from existing toxicological and epidemiological studies;^15,16^ (2) reasonably hypothesized exposure source(s) among our Nicaraguan population;^31,33^ and (3) validation of toenails as an appropriate exposure biomarker.^48,49^ For any samples <LOD, of which there were none in the primary dataset (As, Cd, Hg, Ni, Pb, and U) and few in the full dataset, we used machine-read values. Using directed acyclic graphs (DAGs) informed by existing literature,^50^ we identified sex (M/F), age (continuous), municipality (Chichigalpa, La Paz Centro, and Mina El Limón), and occupational risk (high, low, and nonworker) as factors that may confound the relationships between metal exposures and kidney function (DAG and covariate descriptions in Supplemental Methods; https://links.lww.com/EE/A411). In exploratory analyses, we considered possible metal sources with independent multivariable regressions informed by source-specific DAGs. In these exploratory models, metals were regressed on the following correlates to evaluate direct effects: sex, age, municipality, occupation, drinking water source, and fish consumption.

Generalized additive models (GAMs) with restricted cubic splines (10 knots) were used to visually assess the linearity of relationships between each ln-transformed, *z*-standardized metal and continuous eGFR. Through the visual evaluation of relationship linearity between individual metals and eGFR, GAM results informed the modeling of metal variables in subsequent models. GAMs fit with MGCV R package^51^ were fully adjusted for all six metals (As, Cd, Hg, Ni, Pb, and U) and covariates above.

We also used a more traditional statistical approach of multivariable linear regression to evaluate associations between toenail metal concentrations and continuous eGFR. We used ordinal regression with the polr function in MASS R package to evaluate worsening eGFR categories, delineated above.^52^ Effect estimates from ordinal regression were exponentiated for presentation as odds ratios. We investigated: (1) single-metal models; (2) models coadjusted for all metals of primary interest; and (3) a final, parsimonious model run with any of the six metals consistently associated with eGFR in models 1–2. All regression models were adjusted for sex, age, residential municipality, and occupational risk. We conducted sensitivity analyses with additional adjustment for residential drinking water source, fish consumption, and the previous year’s eGFR. We also explored exclusion of cigarette smokers given a strong sex effect and sparsity of female self-reported smokers (33/36 smokers were male).

We used Bayesian kernel machine regression (BKMR) to examine associations of the metal mixture with continuous eGFR, given its flexible structure and ability to handle highly correlated data, nonlinear exposure-outcome relationships, and higher-order interactions,^53^ which have been reported previously in metal mixture studies.^54–56^ Our final BKMR model is described as:


$$\begin{array}{c} eGFR_{continuous}\sim \;h\left(lnAs_{i},\;lnCd_{i},\;lnHg_{i},\;lnNi_{i},\;lnPb_{i},\;lnU_{i}\right)\\ +\;b_{1}*sex_{i}+b_{2}*age_{i}+b_{3}*municipality_{i}\\ +\;b_{4}*occupational\;risk_{i}+e_{i} \end{array}$$

where the h(•) function represents a flexible kernel exposure-response function of the mixture components,^53^ and *b*’s 1–4 are covariates.

We used the BKMRhat R package^57^ to run three parallel chains with 50,000 iterations each. We used component-wise variable selection and obtained a measure of variable importance for each mixture component (i.e., posterior inclusion probabilities, PIPs). The first 50% of iterations of each chain were used as burn-in for model training and discarded. We used default noninformative priors, evaluated model convergence, and performed sensitivity analyses with different degrees of smoothness and seeds.^58^ We explored a broader mixture effect with the full element panel. All analyses were conducted in RStudio version 2024.12.1.

## Results

The sample of 297 individuals, median age 20 years (IQR 7), included 146 females (49.2%). Among participants, 19.2% (n = 57) reported working in an industry with elevated MeN prevalence (i.e., sugarcane or other agriculture; mining; construction; brick making), versus 28.3% (n = 84) working in industries with low MeN-prevalence (i.e., commerce, mechanics, and childcare). Most participants were students who did not report working full- or part-time (n = 156; 52.5%). Table 1 describes key participant characteristics.

**Table 1.** Distribution of demographic variables

	Sample (n = 297)
Female (n, %)	146 (49.2)
Age (median, IQR)	20 (7)
Municipality (n, %)	
Chichigalpa	118 (39.7)
La Paz Centro	98 (33.0)
Mina el Limon	81 (27.3)
Work status (n, %)	
Nonworker	156 (52.5)
Low-risk worker	84 (28.3)
High-risk worker	57 (19.2)
Consumes fish monthly (n, %)	193 (65.0)
Health conditions (n, %)	
Diabetes	1 (0.33)
Hypertension	3 (1.01)
High BMI	115 (38.7)
Drinking water source (n, %)^a^	
Public supply	242 (86.1)
Well	23 (8.19)
Bottled	16 (5.69)
Ever smoker (n, %)^a^	36 (12.8)
eGFR, ml/min/1.73 m^2^ (median, IQR)	103 (20.2)
eGFR >90 ml/min/1.73 m^2^ (n, %)	221 (74.4)
eGFR 76–90 ml/min/1.73 m^2^ (n, %)	56 (18.9)
eGFR ≤75 ml/min/1.73 m^2^ (n, %)	20 (6.73)

a Data taken from previous year of follow-up questionnaire and missing for 16 participants who were LTF from previous year of cohort follow-up; denominator of 281.

### Kidney function

Median eGFR reflected healthy kidney function (103, IQR 20.2 ml/min/1.73 m^2^). Most individuals had eGFR>90 (n = 221/297, 74.4%), though 18.9% had eGFR 76–90 (n = 56) and 6.73% had eGFR≤75 ml/min/1.73 m^2^ (n = 20).

### Toenail metal concentrations

**Figure 1.**
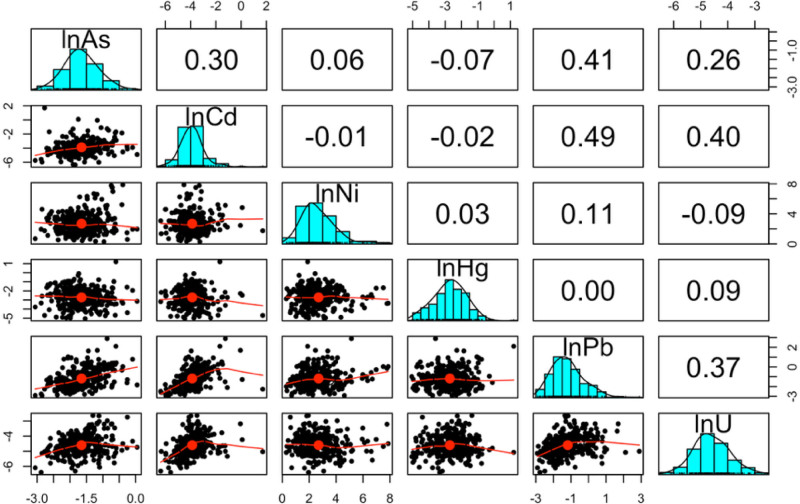
Spearman correlation matrix between the primary nephrotoxic metal mixture.

All toenail samples had concentrations >LOD of the six primary metals (As, Cd, Hg, Ni, Pb, and U). Within the full element set, beryllium and vanadium were excluded for having >60% of samples <LOD and failing to meet quality assurance and quality control criteria, respectively (Table S1; https://links.lww.com/EE/A411). Within the primary metal set, the highest correlations were observed between Pb-Cd (r = 0.49) and Pb-As (r = 0.41; Figure 1). Correlations were higher among metals in the full panel (Figure S1; https://links.lww.com/EE/A411). Tables 2 and S2; https://links.lww.com/EE/A411 provide summary statistics.

**Table 2.** Element concentrations measured in toenails (μg/g) among Jovenes-Nica Study youth (n = 297)

	LOD	% >LOD	Mean (SD)	Geometric mean (mean, SD)	Minimum	25th percentile	Median	75th percentile	Maximum
As	0.032	100	0.22 (0.13)	0.19 (1.69)	0.047	0.14	0.19	0.27	1.05
Cd	0.0005	100	0.056 (0.33)	0.021 (2.68)	0.0017	0.011	0.019	0.032	5.49
Hg	0.003	100	0.11 (0.22)	0.066 (2.72)	0.0064	0.032	0.067	0.13	3.33
Ni	0.159	100	61.1 (250)	14.8 (3.66)	1.34	6.56	11.7	28.0	2614
Pb	0.003	100	0.56 (1.27)	0.31 (2.55)	0.054	0.16	0.28	0.50	18.0
U	0.0002	100	0.013 (0.010)	0.010 (1.94)	0.002	0.0066	0.0094	0.016	0.072

### Metal source investigation

Figure S2; https://links.lww.com/EE/A411 shows individual models built for each predictor of interest. In multivariable models (Table S3; https://links.lww.com/EE/A411), male sex was associated with higher As, lower Cd, and lower U. Age was independently associated with differences in all metal concentrations except Ni. Living in Mina El Limón was associated with higher As and borderline higher Cd, but lower Hg; living in Chichigalpa was associated with lower As, Pb, and U and higher Hg. High-risk jobs were associated with higher As and U. Drinking bottled water was associated with lower Cd. Consuming fish or seafood at least monthly was associated with higher Hg.

### Regression modeling

**Figure 2.**
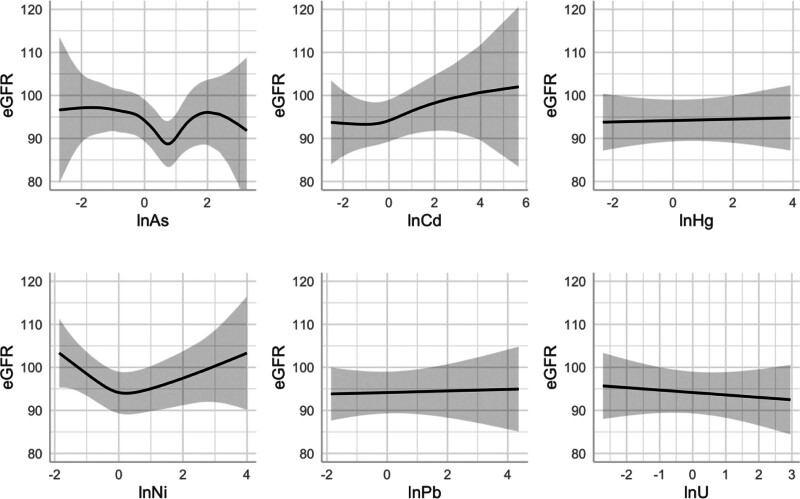
Restricted cubic spline generalized additive models. GAMs display the smoothed relationship between each natural log-transformed, *Z*-standardized metal and continuous eGFR (ml/min/1.73 m^2^). Models were adjusted for the primary metal set (lnAs, lnCd, lnHg, lnNi, lnPb, and lnU) and covariates: sex, age, municipality (Chichigalpa, La Paz Centro, and Mina El Limón), occupational risk group (high risk, low risk, and nonworker).

We used traditional statistical models to simplify interpretability and for more direct comparison to other nonmixture studies. Based on GAMs, associations with continuous eGFR appeared linear for Cd, Hg, Pb, and U, though Ni and As demonstrated *U*-shaped (Ni) or *V*-shaped (As) associations with eGFR (Figure 2). Therefore, we modeled As and Ni as tertiles in subsequent regression models. In adjusted multivariable linear regressions, the middle tertile of Ni and the highest tertile of As were significantly associated with lower eGFR in single-metal models (model 1; As) and both single-metal and copollutant-adjusted models (models 1 and 2; Ni; Table 3). In the most parsimonious model (model 3), the middle tertile of Ni was associated with −6.92 (95% confidence intervals [CI] = −10.7, −3.12) and the highest tertile of As with −4.25 (−8.42, −0.07) ml/min/1.73 m^2^ of eGFR, each compared to the lowest tertiles.

**Table 3.** Linear regression results (beta, 95% confidence intervals) of continuous eGFR as associated with natural log-transformed and *z*-standardized metal concentrations (n = 297)

		Model 1: single metal models	Model 2: fully adjusted	Model 3: coadjusted
As	Lowest	0 (ref)	0 (ref)	0 (ref)
	Middle	−3.03 (−6.94, 0.88)	−2.11 (−6.15, 1.94)	−2.02 (−5.90, 1.87)
	Highest	−**4.71** (−**8.95,** −**0.47**)	−4.58 (−9.29, 0.13)	−**4.25** (−**8.42,** −**0.07**)
Ni	Lowest	0 (ref)	0 (ref)	0 (ref)
	Middle	−**7.17** (−**11.0,** −**3.40**)	−**7.06** (−**10.9,** −**3.24**)	−**6.92** (−**10.7,** −**3.12**)
	Highest	−2.93 (−6.74, 0.87)	−2.83 (−6.67, 1.01)	−2.64 (−6.45, 1.17)
Cd	Continuous	0.81 (−0.83, 2.45)	1.47 (−0.40, 3.34)	
Hg	Continuous	0.06 (−1.60, 1.72)	0.26 (−1.40, 1.92)	
Pb	Continuous	0.00 (−1.66, 1.66)	0.21 (−1.73, 2.15)	
U	Continuous	−0.80 (−2.65, 1.06)	−0.89 (−3.03, 1.24)	

Model 1 is all single metal models; Model 2 is a model coadjusted for all metals; Model 3 is the final, most parsimonious model containing metals significantly associated with eGFR in models 1–2 (As and Ni). All models adjusted for age, sex, residential municipality, and occupational risk category. Model 3 includes only As and Ni, adjusted for covariates. Example interpretation for metals modeled continuously: In Model 1, a one standard deviation increase in natural log-transformed Cd was associated with a 0.81 ml/min/1.73 m^2^ increase in eGFR (95% CI = −0.83, 2.45).

Bolded results indicate statistical significance at alpha = 0.05.

Trends were consistent in ordinal regression. In the most parsimonious model, the middle Ni tertile was associated with 2.34 times the odds of being in a lower eGFR category compared to the lowest Ni tertile (95% CI = 1.19, 4.71; Table 4). The highest tertile of As was also associated with increased odds of lower eGFR compared to the lowest As tertile (odds ratios: 2.05; 95% CI = 1.00, 4.30).

**Table 4.** Ordinal regression results (odds ratios, 95% confidence intervals) of being in a lower eGFR category as associated with natural log-transformed and *z*-standardized toenail metal concentrations (n = 297)

		Model 1: single metal models	Model 2: Fully adjusted	Model 3: coadjusted
As	Lowest	1 (ref)	1 (ref)	1 (ref)
	Middle	1.20 (0.59, 2.43)	1.05 (0.49, 2.22)	1.01 (0.49, 2.08)
	Highest	**2.15 (1.05, 4.48**)	2.23 (0.97, 5.22)	2.05 (1.00, 4.30)
Ni	Lowest	1 (ref)	1 (ref)	1 (ref)
	Middle	**2.29 (1.19, 4.53**)	**2.53 (1.28, 5.17**)	**2.34 (1.19, 4.71**)
	Highest	1.48 (0.73, 3.04)	1.48 (0.72, 3.12)	1.36 (0.67, 2.81)
Cd	Continuous	0.84 (0.62, 1.12)	0.69 (0.46, 1.00)	
Hg	Continuous	1.01 (0.77, 1.34)	1.02 (0.75, 1.38)	
Pb	Continuous	0.96 (0.73, 1.26)	0.91 (0.63, 1.29)	
U	Continuous	1.25 (0.91, 1.73)	1.34 (0.92, 1.96)	

Model 1 is all single metal models; model 2 is a model adjusted for all metals; model 3 is the final, most parsimonious model of metals significantly associated with eGFR in models 1–2. All models adjusted for age, sex, residential municipality, and occupational risk category. Model 3 includes only As and Ni, adjusted for covariates. Example interpretation for metals modeled continuously: In Model 1, a one standard deviation increase in natural log-transformed Cd was associated with 0.84 times the odds of being in a lower eGFR category (95% CI = 0.62, 1.12).

Bolded results indicate statistical significance at alpha = 0.05.

Regression sensitivity analyses controlling for drinking water source (Tables S4 and S5; https://links.lww.com/EE/A411), fish consumption (Tables S6 and S7; https://links.lww.com/EE/A411), and excluding smokers (Tables S8 and S9; https://links.lww.com/EE/A411) were consistent with the main results, though showed slightly attenuated results for As. In models controlling for the past year’s eGFR, the association of As with the same year eGFR was diminished (Tables S10 and S11; https://links.lww.com/EE/A411).

### Bayesian kernel machine regression

**Figure 3.**
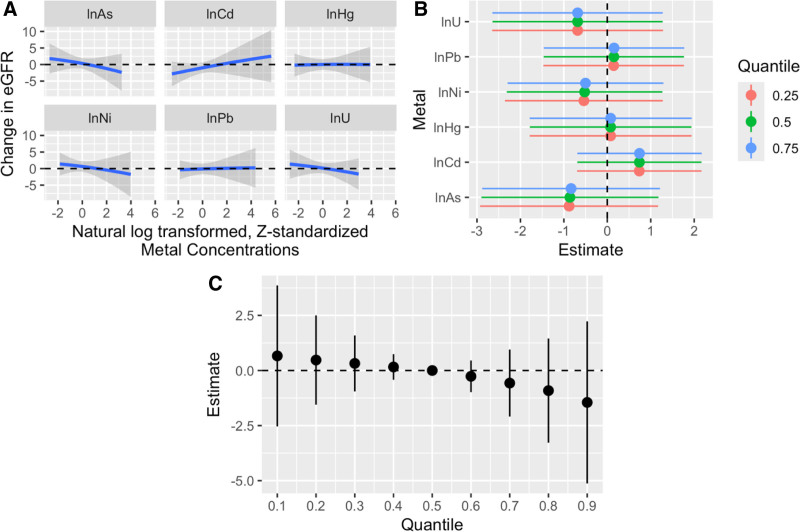
BKMR results adjusted for age, sex, municipality, and occupational risk. A, Univariate predictor responses of the relationship between each metal and the change in eGFR (ml/min/1.73 m^2^) for each standard deviation of exposure when the remaining mixture components are held at their 50th percentile. B, Single variable risk summaries, demonstrating the beta estimates and 95% credible intervals on continuous eGFR of increasing a metal from its 25th to 75th percentile while the rest of the mixture is at 25th, 50th, or 75th percentile—these results investigate higher order interactions. C, Overall mixture effect (betas, 95% credible intervals) on eGFR when the mixture is held at a range of percentiles (10th to 90th) compared to the 50th percentile. Model adjusted for age, sex, residential municipality, and occupational risk category.

**Figure 4.**
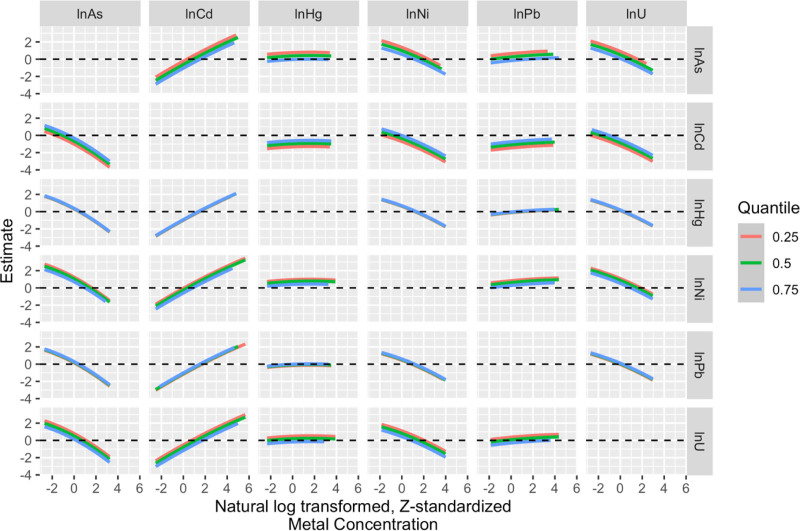
BKMR investigation of pairwise interactions. Bivariate predictor responses show the relationship between metal A (along the columns) and eGFR when metal B (in the rows) is held at 25th, 50th, or 75th percentiles and the remaining mixture components are held at their 50th percentile. These results investigate pairwise metal–metal interactions that may contribute to any observed higher-order interactions. Model adjusted for age, sex, residential municipality, and occupational risk category.

We used BKMR to explore any nonadditive mixture effect in addition to higher-order interactions. Trace, density, and autocorrelation plots indicated model convergence, and Gelman-Rubin statistics for covariates were <1.05 (Figures S3–S5; https://links.lww.com/EE/A411 and Table S12; https://links.lww.com/EE/A411). While Ni and As contributed most to the associations with eGFR (Table S13; https://links.lww.com/EE/A411; PIPs: 0.07 and 0.03, respectively, with the four other metals below 0.02), the overall mixture association with eGFR was weak and largely null (Table S14; https://links.lww.com/EE/A411, Figure 3C). However, some associations were seen for individual metals when assessed in the context of the mixture: while holding all other mixture components at the 50th percentile, Ni, As, and U were weakly negatively and Cd weakly positively associated with eGFR (Figure 3A). There was no visual evidence of nonlinear relationships between any metals and eGFR and no evidence of pairwise or higher-order interactions (Figures 3B and 4).

Evaluation of the full element set showed an overall null mixture effect like that of the primary analysis with six metals, as Ni had the highest PIP (Diagnostics in Figures S6–S8; https://links.lww.com/EE/A411 and Table S15; https://links.lww.com/EE/A411; Results in Figure S9; https://links.lww.com/EE/A411 and Table S16; https://links.lww.com/EE/A411). Sensitivity analyses with different seeds and beta were consistent with primary analyses (Figures S10–S12; https://links.lww.com/EE/A411 in the Supplementary Discussion).

## Discussion

Within our sample of adolescents and young adults living in high-MeN-prevalence regions of Nicaragua, As and highly elevated Ni toenail concentrations were consistently associated with lower eGFR. We found little evidence for a joint effect of a mixture of six metals (Ni, As, Cd, Hg, Pb, and U), metal–metal interactions, or higher-order interactions using BKMR. Our results support independent associations between Ni and As and reduced eGFR, but do not suggest that these metals interact or have greater than additive effects on eGFR.

### Nickel

We observed strikingly elevated concentrations of toenail nickel (median 11.7; Q1, Q3 6.53, 28.3 µg/g). Ni toenail concentrations in this study were >16 times higher than the median Ni concentrations identified in the only other MeN-relevant evaluation of toenail metals (median 0.70 mg/kg; Q1, Q3 0.15, 2.04), which also used IC-PMS analysis.^6^ Compared to toenails analyzed in the same laboratory, our Ni concentrations were 29 times higher than a US adult women’s cohort (median 0.4; Q1, Q3 0.2, 1.1 µg/g)^59^ and five times higher than a Bangladeshi youth cohort (median 2.76 µg/g; IQR 2.29), though in the same magnitude as nails from a Chicago youth cohort (ages 7–15 years) living in an industrial corridor (median 13.4 µg/g; IQR 35.4).^60^ In an assessment of MeN-relevant AKI-risk within a small sample of Nicaraguan sugarcane workers (n = 50), Fischer et al^6^ identified higher Ni among AKI cases compared to controls (univariate test, *P* < 0.001); no other elements in their 15-element panel were associated with AKI. Four other studies evaluating metal exposure and MeN found no significant association between Ni and kidney health, though the concentrations in these studies are not directly comparable given their use of different biomarkers and possible differences in exposure timing and participant characteristics.^37–40^

The kidney is the major target of Ni toxicity, where Ni exposure has been shown to damage mitochondria and increase oxidative stress.^22,61^ Ni has been identified as a nephrotoxicant across animal and epidemiological studies, some of which account for temporality through a prospective design.^62,63^ Inhalation and ingestion of contaminated air, food, and water are major Ni exposure pathways.^55,64^ Ni pollution is most often due to industrial activities including electroplating, electroforming, and battery production, as well as coal combustion, waste incineration, and weathering of geological formations.^64^ Our study was not directly designed to identify exposure sources, and exploratory analyses did not find any covariates associated with Ni concentrations (Table S3; https://links.lww.com/EE/A411), though our elevated exposure levels support future source exploration.

Our identification of a *U*-shaped curve between Ni and eGFR suggests that different Ni exposure levels may be associated with reduced eGFR or hyperfiltration. These findings complement work by Nan et al,^55^ who detected a nonlinear, inverted *U*-shaped dose-response between urine Ni and eGFR among US adults. Given that urine excretion decreases circulating serum levels of metabolites, which source keratin formation in the toenails, we may expect the relationship between kidney function and urinary biomarkers to be inverse of the relationship between kidney function and toenail biomarkers. Reverse causation likely does not fully explain Ni’s *U*-shaped dose response curve; toenails are generally less susceptible to impacts of current kidney function as they reflect the past 7−12 months of metal exposure,^48^ and the association between Ni and eGFR remained significant when adjusting for the previous year’s eGFR (Tables S10 and S11; https://links.lww.com/EE/A411). The Supplementary Discussion provides a deeper discussion of hyperfiltration and forward and reverse causation in the context of our data. It remains possible that random error in this relatively modest sample or residual confounding by an unmeasured confounder partially or fully explains our observed *U*-shaped response curve. It is also feasible that the *U*-shaped curve indicates a threshold effect within our Ni exposure range. Epidemiological evidence for Ni nephrotoxicity is not entirely consistent across single- or co-exposure studies; interactions between Ni and metals or other nephrotoxicants unaccounted for in this study may differ at high Ni concentrations, which could also contribute to the observed *U*-shaped curve. Despite these limitations, our analysis supports further investigation of Ni nephrotoxicity at different exposure concentrations.

### Arsenic

Arsenic is a toxic and carcinogenic metalloid with well-known nephrotoxicity^65^ likely through the generation of reactive oxygen species, genotoxicity, and oxidative stress.^15,16^ Arsenic occurs naturally in the Earth’s crust, and ingestion of contaminated water is the most common exposure route;^15^ industrial activities, mining, and agrichemical production also lead to air contamination.^66^ Toenail As concentrations in our cohort (median 0.18 µg/g; 0.13–0.27) were generally similar to or lower than published concentrations in other international research.^67^ Our concentrations were comparable to those evaluated in Fischer et al’s^6^ occupational cohort (median 0.16 mg/kg; Q1–Q3 0.08, 0.33) and among youth living in a highly exposed industrial corridor near Los Angeles, United States (median 0.16 µg/g; Q1–Q3 0.10, 0.29).^68^ Arsenic has been identified in groundwater^31^ and well water^32^ across northwestern Nicaragua, although analysis of water in our study regions along the Pacific coast has been scarce. In our cohort, As concentrations were elevated among males and high-risk workers, suggesting a potential occupational exposure source. Residents of Mina El Limón, a region with concentrated mining activity, had the highest As toenail concentrations. Sensitivity analyses excluding smokers showed attenuated associations, potentially indicating cigarette smoking as an important confounder of the relationship with eGFR, although our small sample of smokers limits our ability to evaluate this relationship. We expect residual confounding to explain the nonlinear relationship between As and eGFR suggested in GAMs, given that adjustment for fish consumption, smoking status, and the previous year’s eGFR attenuated As results in regressions.

Associations between As and kidney endpoints are inconsistent within the MeN literature and beyond.^18,69–71^ Similar to our findings, Butler-Dawson et al^37^ showed a negative relationship between urinary As and eGFR (−4.36, 95% CI = −7.07, −1.64) in a cross-sectional study of Guatemalan sugarcane workers. McClean et al also reported decreased eGFR (9.0 ml/min/1.73 m^2^) between Nicaraguan sugarcane workers with the highest 10th percentile of urinary As exposure compared to those in the bottom 90th percentile (*P* = 0.01), though this did not hold in covariate-adjusted analyses.^39^ Additionally, Bustamante-Montes et al^38^ found hair As positively associated with CKD risk in a Mexican case-control study. In contrast, Smpokou et al^40^ and Fischer et al^6^ found no association between kidney health and total urinary As or toenail As, respectively, in Nicaraguan populations. Amid varied MeN-relevant findings, our detection of low-level toenail As associated with lower eGFR in young people indicates that As exposure in Central America remains an important avenue of investigation.

### Evaluation of other metals

Besides Ni and As, no other metals of primary interest (Cd, Hg, Pb, and U) were associated with kidney function. Cd showed visual evidence of a weak, positive linear relationship with eGFR in GAMs and BKMR and only a marginal positive association in regression models. These Cd results may be partially or fully explained by residual confounding (especially by smoking status) and/or the incomplete capture of Cd through toenail biomarkers. While toenail As and Hg have been strongly validated against other biological and environmental markers, Cd, Ni, Pb, and U have been less so.^48,49^ It also is possible that metals besides As and Ni are not nephrotoxic at the levels observed in our population. Though Butler-Dawson et al^37^ found urinary Cd as negatively associated with eGFR and positively associated with the kidney injury biomarker, Neutrophil Gelatinase Associated Lipocalin , no other MeN studies found a relationship between Cd, Hg, Pb, or U with kidney health. Exposure to As,^36^ Pb,^36,72^ and Cd^73^ has been more strongly hypothesized to increase CKDu risk in Sri Lanka,^35^ although these associations have not been replicated in MeN studies.

### Evaluation of a mixture effect

We found little evidence of an overall mixture effect. BKMR analyses suggested generally null associations and did not replicate the findings of nonlinearity for Ni and As detected in GAMs and regression analyses. These differences may reflect a relatively small sample size as well as differences in model structure. The BKMR h(•) function operates on different statistical assumptions than linear regression. Namely, BKMR univariate dose response curves show the relationship between eGFR and each metal as a component of the entire mixture within the Gaussian kernel (h) function, rather than marginal associations reflected by individual regression coefficients. In the absence of a joint mixture effect, GAMs that model nonjoint, exposure-specific effects may provide a more appropriate representation of these relationships.

Our detection of highly elevated Ni concentrations in young people may reflect an important avenue of early life nephrotoxic insult, which we hypothesize may increase disease susceptibility in later life. Prenatal and early life metal exposures are associated with increased renal and cardiovascular injury and subsequent disease risk in later life.^74,75^ There is notably less research on these relationships during adolescence, despite continued development of renal and other body systems during these years, allowing for vulnerability to insult. Given the suspected multiplicity of exposures associated with full MeN presentation, nephrotoxic injury during early life and adolescence could be an important disease predictor in conjunction with later life or occupational exposures.

### Limitations

Reverse causation is an important consideration of any biomonitoring work related to kidney function outcomes, especially given the cross-sectional nature of this study,^76^ though toenail biomarkers are likely less confounded by current kidney health status than urine or serum biomarkers (see Supplementary Discussion; https://links.lww.com/EE/A411). It is possible that we were unable to accurately detect or describe small effects or higher-order interactions given our relatively small sample size. Given the low prevalence of reported smoking, dichotomous smoking responses (ever/never) from self-reports, and a sparsity of female smokers, we were unable to fully evaluate smoking status as a metal source or confounder. Sensitivity analysis excluding smokers attenuated As results (Tables S8 and S9; https://links.lww.com/EE/A411), suggesting confounding of this relationship, although no other metal associations appeared affected. Metal contamination by toenail clippers or superficial contamination of toenails (e.g., nail polish, dirt) may partially explain our findings of elevated Ni concentrations, especially since some nail clippers may be Ni-plated; although we followed established and published collection protocol for toenail metals assessment,^60^ it is possible that exogenous contamination existed. Field-based cleaning with alcohol wipes and validated nail cleaning protocols (Supplemental Methods; https://links.lww.com/EE/A411) are believed to address most contamination from nail polish or dirt, although external contamination may have remained. The lack of As speciation limits our ability to describe organic As, which can be sourced from seafood in the diet; As was not associated with fish consumption in our metal source investigation (Table S3; https://links.lww.com/EE/A411), though increased Hg was, which helps validify our model and supports a lack of identifiable association between total toenail As and seafood. Nondifferential exposure misclassification is possible and may have attenuated associations. Amid varying evidence for the applicability of toenails as metal biomarkers, including for Ni, it is possible that there is nondifferential exposure misclassification;^77^ future investigation of other metal biomarkers is an important next step. While our study population was focused on identifying early life exposures and kidney outcomes specifically among high-risk youth in Nicaragua, it may be of limited generalizability to occupational cohorts, individuals with MeN, or older adults. We were unable to fully identify sources of metals exposure in our population and suggest this as an important area for follow-up investigation regarding Ni and As.

### Strengths

The use of toenail biomarkers to reflect historical exposure is an important strength, as our associations may be less confounded by reverse causation. Our use of IC-PMS allowed for low detection limits and gold-standard exposure classification. While arsenic was not speciated, inorganic As has higher nephrotoxic potential and is more likely to be keratinized in toenail tissue than organic As.^67^ The European Kidney Function Consortium eGFR equation has been validated in young cohorts across North America, Europe, and Africa and across the adolescent age range, which is a strength for our purposes.^44–46^ Our use of BKMR allowed for the investigation of higher-order interactions and joint effects, and our conclusions are strengthened by consistency across multiple traditional regression models. The inclusion of females and nonworking young people reflects understudied populations in MeN research.^33^

## Conclusions

Elevated Ni and As toenail concentrations were associated with low eGFR in this study of Nicaraguan adolescents in areas with high MeN prevalence. We did not observe associations between other metals and kidney outcomes, nor a multiplicative mixture effect. Our findings suggest a need to validate findings among other biological biomarkers and for further targeted efforts to identify sources of these metals, especially Ni, which we observed at extremely elevated concentrations; investigation of occupational and residential exposure to mining operations, roofing or housing materials, water source and diet are important areas for source exploration. We are unable to establish causal evidence regarding metal exposure and MeN in this cross-sectional analysis, as no participants are MeN cases. However, evidence of elevated Ni concentrations among at-risk young people supports the need for further targeted metal surveillance in Central America and considerations of early life metal exposure as a CKDu susceptibility factor.

## Conflicts of interest statement

The authors declare that they have no conflicts of interest with regard to the content of this report.

## ACKNOWLEDGMENTS

*To all the Estudio Jóvenes Nica participants and their families, as well as the Nicaraguan field team, especially Josefa Elena Moya González — a large and lasting thank you. We are also grateful to Stephanie Gonzalez Gil for her contributions to this study.*

## Supplementary Material
