# Supplementary material for: Toenail metal concentrations and kidney health among young people at risk of chronic kidney disease of uncertain etiology
Source: Environ Epidemiol. 2026 Feb 9;10(2):e461. doi: 10.1097/EE9.0000000000000461 (PMC12889388; doi:10.1097/EE9.0000000000000461)
Supplement: Supplementary file 1 [file ee9-10-e461-s001.pdf]

SUPPLEMENTAL INFORMATION: Toenail metal concentrations and kidney health among young people at risk of chronic kidney disease of uncertain etiology (CKDu)

Table of Contents

I. **Supplemental Methods:** Directed Acyclic Graph (DAG), covariate descriptions, and laboratory specifics
II. **Table S1:** QA/QC Summarized for full element panel
III. **Figure S1:** Spearman correlation plots of the entire element panel
IV. **Table S2:** Summary statistics for metals of secondary interest.
V. **Figure S2:** Source specific DAGs used to build models
VI. **Table S3:** Source-specific models assessing direct effects of each specific covariate, based on source-specific DAGs.
VII. **Table S4:** LINEAR regressions with drinking water source added as a covariate
VIII. **Table S5:** ORDINAL regressions with drinking water source added as a covariate
IX. **Table S6:** LINEAR regressions with monthly fish consumption added as a covariate
X. **Table S7:** ORDINAL regressions with monthly fish consumption added as a covariate
XI. **Table S8:** LINEAR regressions EXCLUDING those who ever smoked (n=245)
XII. **Table S9**: ORDINAL regressions EXCLUDING those who ever smoked (n=245)
XIII. **Table S10**: LINEAR regressions adjusting for previous year's eGFR
XIV. **Table S11**: ORDINAL regressions adjusting for previous year's eGFR
XV. **Figure S3:** Trace plots from BKMRhat and coda packages between the assessment of continuous eGFR and the primary metal mixture (As, Cd, Hg, Ni, Pb, U).
XVI. **Figure S4:** BKMR Autocorrelation plot from the combined kmfit for the primary metal set
XVII. **Figure S5:** Cross correlation plot from the combined kmfit for the primary metal set (after combined all 3 chains)
XVIII. **Table S12:** Diagnostics of BKMR ran with 3 chains and 50k iterations/chain on the PRIMARY metal set.
XIX. **Table S13:** Posterior inclusion probabilities (PIPs) from BKMR of the primary metal set.
XX. **Table S14:** Effect estimates from BKMR of the overall metal mixture (lnAs, lnCd, lnHg, lnNi, lnPb, lnU) with eGFR when all components are held at different percentiles, compared to when all components are at their 50th percentiles.
XXI. **Figure S6:** Trace plots from BKMRhat between the assessment of continuous eGFR and the full element mixture, n=20
XXII. **Figure S7:** BKMR Autocorrelation plots from BKMRhat between the assessment of continuous eGFR and the full element mixture, n=20

XXIII. **Figure S8:** BKMR Crosscorrelation plots from BKMRhat between the assessment of continuous eGFR and the full element mixture, n=20
XXIV. **Table S15:** Diagnostics of BKMRhat between the assessment of continuous eGFR and the full element mixture, n=20
XXV. **Figure S9:** Results (univariate relationships and overall mixture effect) from BKMR ran between the assessment of continuous eGFR and the full element mixture, n=20
XXVI. **Table S16:** PIPs from BKMR with complete element set, n=20
XXVII. **Supplementary Discussion:** Metal biomarkers and applications to forward and reverse causality

Supplemental Methods: Directed Acyclic Graph (DAG), covariate descriptions, and laboratory specifics

DAG modeling a direct effect of toenail metal concentration against serum creatinine-based eGFR. Dashed lines indicate less-well supported relationships or ones that we were statistically underpowered to assess here, and those covariates (drinking water source, fish consumption, cigarette smoking) were evaluated in sensitivity analyses.

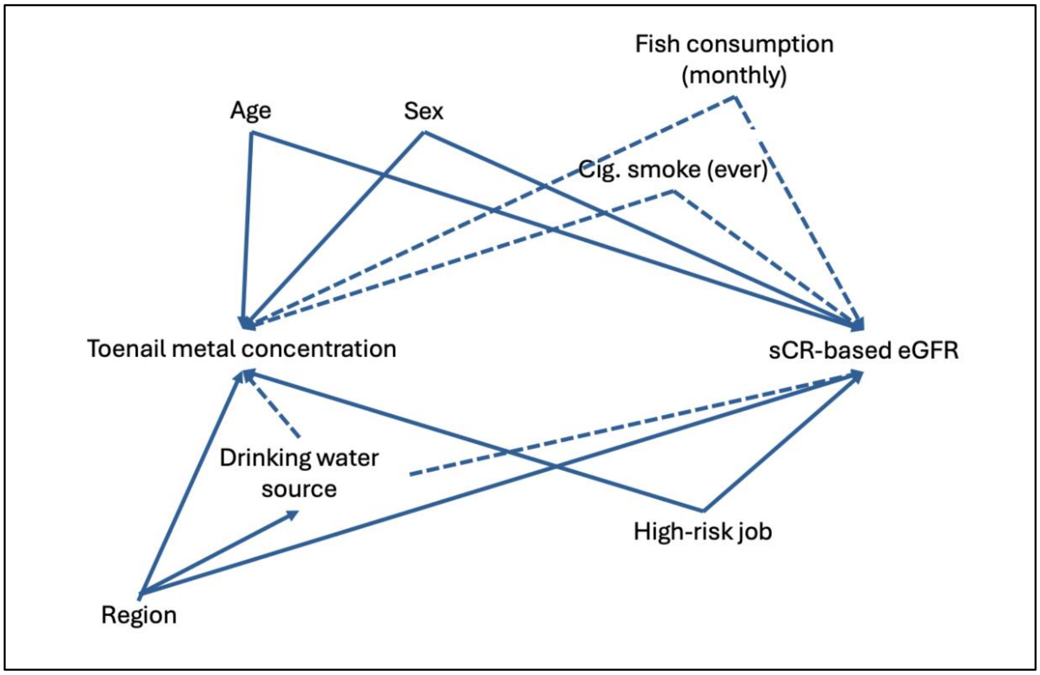

Covariate Descriptions

- Risk category of current occupation: From oral report of current industry and job task, we created a variable assigning risk level to each participant's occupation, as informed by our previous work: high risk, low risk, or nonworker. Jobs that could be both a metal exposure source and associated with other factors potentially (i.e. high heat or strenuousness of activities) were considered high risk; we identified working in agriculture, brick making, mining, and construction as high risk. All other occupations were considered low risk. Individuals not working full or part time were considered nonworkers.
- Residential drinking water source: Drinking water source was a factored variable of public drinking water (from Empresa Nicaragüense de Acueductos y Alcantarillados Sanitatios, ENACAL), wells (communal or personal) and bottled water.
- Smoking status:

- Fish consumption: Self-report of shellfish or seafood consumption, as ever consumed in last month vs not

ICP-MS Analysis

Inductively coupled plasma mass spectrometry (ICP-MS) was conducted at the Dartmouth College Trace Element Analysis Core. There, dirt and debris were removed from the toenails in preparation for washing, which occurred in a 7mL polyethylene vial. The washing steps are as follows: 5 washes with 2mL of acetone (ACS grade) and a 20-minute ultrasonic bath; 5 washes with 2mL of 1% Trito X-100 solution and a 20-minute ultrasonic bath; 5 washes with 2mL of MilliQ deionized water. Washing steps were followed by drying in a dry box. When dried, samples are placed into 15mL vials for weighing and digestion before analysis.

Digestion steps are as follows: cold digestion of at least 12 hours with 0.5 or 1 mL of HNO_3/HCl (as a 9:1 ratio); microwave digestion after adding 100 or 200 uL of trace metal grade H_2O_2. During microwave digestion, samples are heated to 105C across 15 minutes and held there for 45 minutes. After digestion, samples are diluted to with MilliQ deionized water to 5 or 10 mL (depending on the initial sample mass and acid addition) and weighed. Ten replicate reference materials were included per digestion batch of 100 samples; 5 each of ERM DB001 (Human hair, European Reference Material, Geel, Belgium) and IAEA 086 (Human hair, International Atomic Energy, Vienna, Austria).

Prepared samples are processed by ICP-QQQ-MS (Aglient 8900 Wilmington, DE) using a prepFAST M5 autosampler. NIST-traceable custom element standards are used to prepare primary standards and NIST-traceable second source calibration standards are used to prepare the Internal Calibration Verification (ICV), which is run after every 10 samples.

We utilized the median detection limit (MDL) as the limit of detection (LOD) in this analysis (see main text, Methods section). The MDL is calculated by taking the median of the detection limits for all samples of a single element. Calculating the MDL is advantageous as it accounts for the range of dilution factors present across a sample; dilution factors impact samples with varying masses differently (i.e. samples with higher mass may have lower individual detection limits, even between samples with the same actual mass-corrected concentration of an element). Because our toenail samples had a wide range of masses, utilization of the MDL was most appropriate.

TABLE S1: QA/QC Summarized for full element panel.

	Duplicate			Referent (hair)			Spike			
Analyte	N	Min, max RPD	Mean RPD	N	Min, max % rec	Mean % rec	N	Min, max % rec	Mean % rec	Passed
Ag	13	0.43, 102	19.1	0			15	10.2, 104	95.8	
Al	13	1.47, 25.7	12.2	0			1	27.83	27.8	
As	13	0.07, 18.7	6.09	15	97.3,136.6	113	15	7.00, 105	86.4	
Ba	13	0.04, 9.16	4.91	0			2	4.01, 103	53.6	
Be	0			0			15	27.0, 116	97.4	Excluded
Cd	13	3.40, 39.4	17.0	15	76.6,126.4	88.0	15	27.6, 111	98.6	
Co	13	7.29, 20.7	15.8	0			15	13.78, 142	119	
Cr	11	0.89, 12.1	6.12	0			12	11.5, 105	88.0	
Cu	13	0.14, 5.48	2.74	31	89.3, 703	117	1	2.57	2.57	
Fe	13	3.43, 15.6	12.9	16	77.5, 173	89.6	1	13.14	13.1	
Hg	13	1.19, 31.8	11.2	31	69.1, 112	81.6	13	5.36, 89.0	77.9	
Mn	13	2.30, 17.3	13.8	16	92.7, 162	104	3	13.6, 134	90.5	
Mo	13	0.41, 38.8	16.3	0			15	17.9, 116	90.6	
Ni	13	1.63, 16.5	11.5	0			2	7.95, 125	66.5	
Pb	13	0.72, 27.9	8.06	15	82.3, 152	93.9	15	8.76, 127	103	
Sb	13	0.82, 13.9	5.17	0			12	2.94, 107	85.3	
Se	13	0.42, 12.9	7.17	31	71.7, 94.1	82.4	15	7.04, 104	83.7	
Sn	13	0.12, 9.72	4.89	0			15	5.47, 268	111	
Sr	13	3.88, 18.9	11.0	0			5	16.2, 108	82.3	
Tl	13	0.00, 26.1	16.6	0			15	8.31, 106	96.5	
U	13	0.46, 30.0	15.1	0			15	19.1, 112	102	
V	13	7.72, 26.8	21.0	0			15	24.0, 502	176	Excluded
Zn	13	0.79, 18.4	9.48	31	73.9, 101	83.4	1	10.1	10.1	

RPD= relative percent difference

Nickel was not certified in either standard reference material (SRM), however Informational value for ERM DB 001 lists Ni concentration as 0.78 µg/g compared with our result of 1.34 ± 0.57 µg/g (N = 15). Literature values for IAEA086 are reported in the on-line database Georem to be 2.4-3.04 µg/g based on 3 values compared with our result of 3.34 ± 0.24 µg/g (N = 15, one outlier excluded).

FUGURE S1: Spearman correlation plots of the entire element panel (removing Be and V for having>60% of samples <LOD and for failing QA/QC standards).

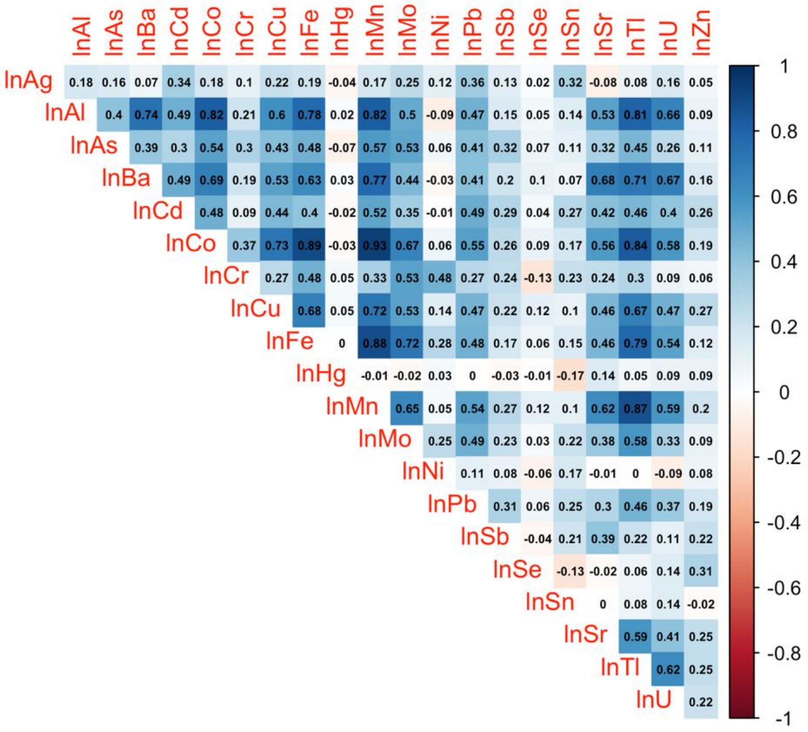

TABLE S2: Summary statistics for metals of secondary interest.

	LOD	% <LOD	Mean (SD)	Minimum	25th percentile	Median	75th percentile	Maximum
**Ag**	0.0011	0.33	0.30 (3.69)	0.00	0.01	0.01	0.03	62.0
**Al**	6.3934	0	377 (401)	18.9	142	247	460	3750
**Ba**	0.0082	0	10.5 (15.5)	0.92	3.83	6.13	11.4	147
**Be**	0.0069	90.3	0.00 (0.00)	0.00	0.00	0.00	0.00	0.02
**Co**	0.0014	0	0.17 (0.21)	0.01	0.06	0.11	0.21	2.45
**Cr**	0.1078	5.67	0.91 (2.99)	0.01	0.21	0.34	0.56	34.0
**Cu**	0.0335	0	4.99 (4.97)	1.16	3.57	4.26	5.30	82.0
**Fe**	8.2297	0	686 (1670)	26.1	178	306	549	18700
**Mn**	0.0127	0	11.6 (16.7)	0.73	3.59	6.52	14.0	217
**Mo**	0.0012	0	0.03 (0.076)	0.00	0.01	0.02	0.03	0.99
**Sb**	0.0030	0	0.10 (0.14)	0.01	0.03	0.06	0.11	1.50
**Se**	0.0305	0	0.71 (0.15)	0.18	0.64	0.72	0.79	1.63
**Sn**	0.0421	3.00	2.02 (7.09)	-0.02	0.12	0.22	0.50	66.6
**Sr**	0.0154	0	3.63 (2.69)	0.45	1.97	3.02	4.52	22.5
**Tl**	0.0002	1.00	0.00 (0.00)	0.00	0.00	0.00	0.00	0.01
**V**	0.0023	0	0.92 (1.26)	0.06	0.32	0.55	1.09	16.3
**Zn**	0.4015	0	111 (109)	24.5	75.3	85.7	99.3	817

FIGURE S2. Source specific DAGs used to build models. Variables in red show the direct effect under investigation in each model.

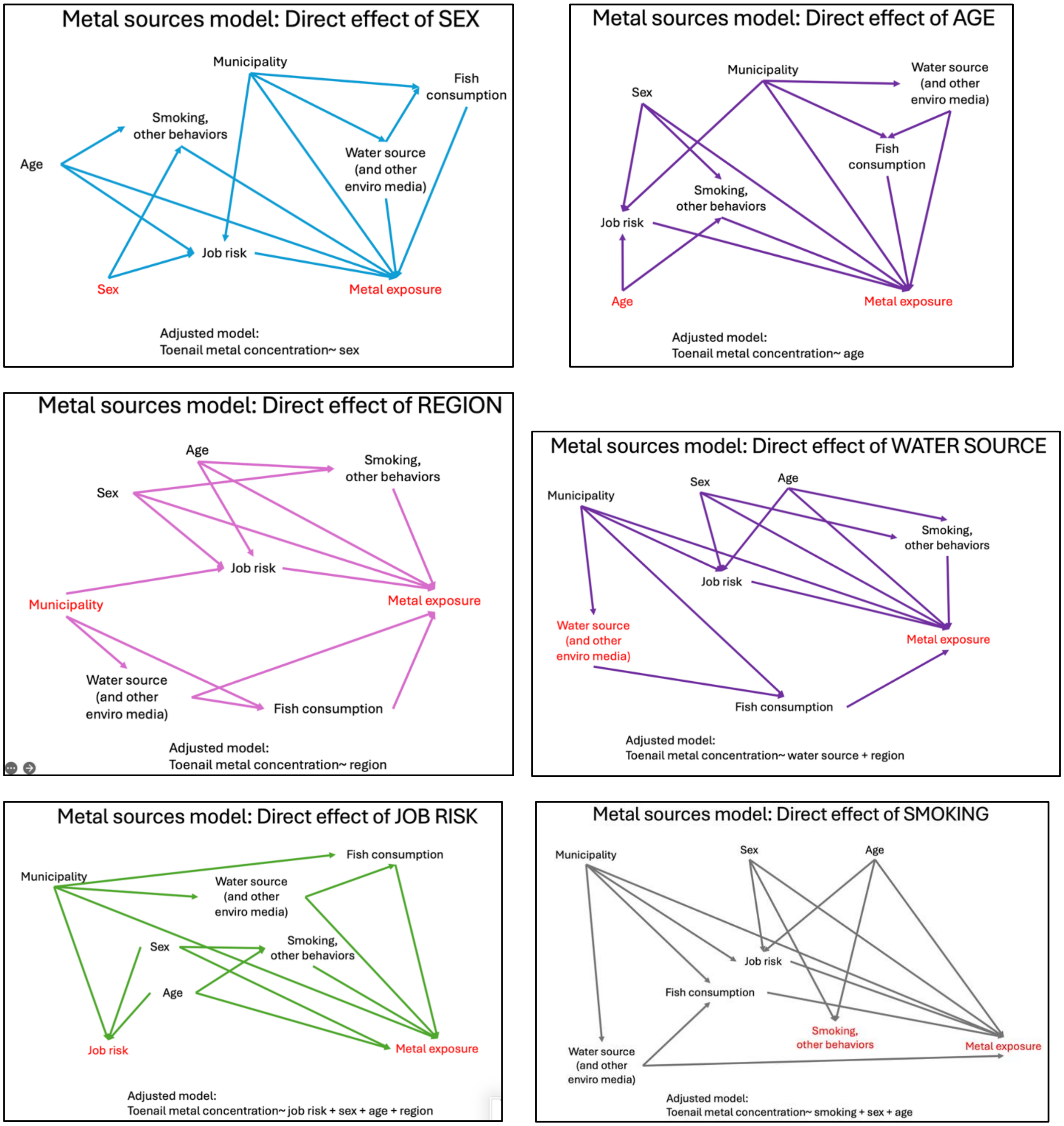

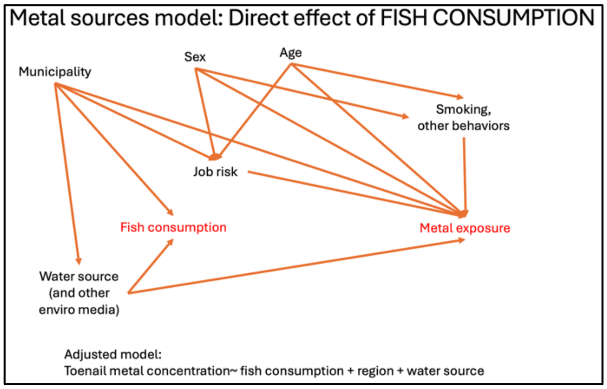

Metal sources model: Direct effect of FISH CONSUMPTION
Sex
Age
Municipality
Smoking,
other behaviors
Job risk
Fish consumption
Metal exposure
Water source
(and other
enviro media)
Adjusted model:
Toenail metal concentration~ fish consumption + region + water source

TABLE S3. Source-specific models assessing direct effects of each specific covariate, based on source-specific DAGs. Variable definitions included in main Methods section; beta estimates and 95% CIs were back-transformed through exponentiation. Bolded results show statistical significance at alpha=0.05.

	Arsenic	Nickel	Cadmium	Mercury	Lead	Uranium
**Male sex[a]**	**1.29 (1.15, 1.45)**	0.85 (0.64, 1.15)	**0.69 (0.56, 0.87)**	1.00 (0.79, 1.24)	0.95 (0.77, 1.18)	**0.82 (0.71, 0.95)**
**Age[b]**	**0.97 (0.96, 0.98)**	1.00 (0.97, 1.04)	**0.95 (0.93, 0.98)**	**1.06 (1.03, 1.09)**	**0.93 (0.91, 0.96)**	**0.97 (0.96, 0.99)**
**Job Risk[c]**						
**Nonworker**	Ref	Ref	Ref	Ref	Ref	Ref
**Low risk**	1.00 (0.86, 1.15)	0.74 (0.50, 1.01)	1.05 (0.78, 1.40)	0.78 (0.58, 1.05)	0.95 (0.73, 1.25)	0.94 (0.79, 1.12)
**High risk**	**1.27 (1.08, 1.49)**	0.77 (0.49, 1.21)	1.12 (0.80, 1.57)	0.98 (0.70, 1.37)	0.98 (0.72, 1.34)	**1.37 (1.12, 1.68)**
**Municipality[d]**						
**La Paz Centro**	Ref	Ref	Ref	Ref	Ref	Ref
**Chichigalpa**	**0.77 (0.68, 0.88)**	1.10 (0.78, 1.56)	0.97 (0.75, 1.27)	**1.32 (1.02, 1.71)**	**0.72 (0.57, 0.92)**	**0.66 (0.56, 0.77)**
**Mina el Limón**	**1.24 (1.07, 1.43)**	1.44 (0.98, 2.11)	1.32 (0.99, 1.76)	**0.65 (0.49, 0.86)**	1.21 (0.92, 1.58)	**0.47 (0.40, 0.57)**
**Water source[e]**						
**ENACAL**	Ref	Ref	Ref	Ref	Ref	Ref
**Bottled**	0.83 (0.65, 1.06)	1.45 (0.73, 2.88)	**0.55 (0.33, 0.92)**	1.27 (0.76, 2.10)	0.81 (0.50, 1.32)	1.04 (0.76, 1.42)
**Well**	1.10 (0.90, 1.34)	0.96 (0.55, 1.68)	0.91 (0.60, 1.39)	1.43 (0.94, 2.17)	0.80 (0.54, 1.19)	1.24 (0.96, 1.60)
**Fish consumption[f]**	1.01 (0.90, 1.14)	0.96 (0.70, 1.33)	0.92 (0.72, 1.17)	**1.98 (1.58, 2.49)**	1.05 (0.84, 1.33)	1.03 (0.89, 1.19)

[a] Model ran to evaluate sex as a dependent variable: lnMetal ~ sex
[b] Model ran to evaluate age as a dependent variable: lnMetal ~ age
[c] Model ran to evaluate male job as a dependent variable: lnMetal ~ job + sex +age + municipality (compared to nonworkers)
[d] Model ran to evaluate residential municipality ("region" in Figure S2) as a dependent variable: lnMetal ~ municipality (compared to La Paz Centro)
[e] Model ran to evaluate drinking water source as a dependent variable: lnMetal ~ water source (compared to ENACAL) + municipality
[f] Model ran to evaluate drinking water source as a dependent variable: lnMetal ~ fish consumption + water source+ municipality

TABLE S4. LINEAR regressions with drinking water source added as a covariate. Models adjusted for sex, age, residential municipality, occupational risk category, and drinking water source.

		Model 1: Single metal models	Model 2: Fully adjusted	Model 3: Co-adjusted
As	Lowest	0 (ref)	0 (ref)	0 (ref)
	Middle	-3.48 (-7.51, 0.55)	-2.64 (-6.80, 1.52)	-2.40 (-6.40, 1.59)
	Highest	**-5.07 (-9.44, -0.69)**	**-5.02 (-9.87, -0.16)**	**-4.38 (-8.70, -0.06)**
Ni	Lowest	0 (ref)	0 (ref)	0 (ref)
	Middle	**-7.69 (-11.6, -3.77)**	**-7.37 (-11.3, -3.39)**	**-7.29 (-11.2, -3.35)**
	Highest	-3.75 (-7.68, 0.18)	-3.37 (-7.36, 0.62)	-3.27 (-7.22, 0.68)
Cd	Continuous	0.73 (-0.98, 2.44)	1.33 (-0.62, 3.29)	
Hg	Continuous	0.26 (-1.47, 1.98)	0.44 (-1.28, 2.17)	
Pb	Continuous	-0.11 (-1.82, 1.60)	0.11 (-1.91, 2.13)	
U	Continuous	-0.47 (-2.40, 1.47)	-0.46 (-2.68, 1.77)	

TABLE S5. ORDINAL regressions with drinking water source added as a covariate. Models adjusted for sex, age, residential municipality, occupational risk category, and drinking water source.

		Model 1: Single metal models	Model 2: Fully adjusted	Model 3: Co-adjusted
As	Lowest	1 (ref)	1 (ref)	1 (ref)
	Middle	1.22 (0.59, 2.52)	1.06 (0.49, 2.30)	1.02 (0.48, 2.15)
	Highest	**2.20 (1.04, 4.71)**	2.29 (0.97, 5.51)	2.03 (0.95, 4.39)
Ni	Lowest	1 (ref)	1 (ref)	1 (ref)
	Middle	**2.44 (1.22, 5.04)**	**2.66 (1.29, 5.68)**	**2.46 (1.21, 5.17)**
	Highest	1.73 (0.83, 3.67)	1.70 (0.80, 3.73)	1.56 (0.74, 3.35)
Cd	Continuous	0.86 (0.62, 1.17)	0.73 (0.48, 1.08)	
Hg	Continuous	0.96 (0.71, 1.28)	0.96 (0.70, 1.32)	
Pb	Continuous	0.97 (0.73, 1.28)	0.90 (0.61, 1.30)	
U	Continuous	1.17 (0.84, 1.64)	1.25 (0.84, 1.87)	

TABLE S6. LINEAR regressions with monthly fish consumption added as a covariate. Models adjusted for sex, age, residential municipality, occupational risk category, and monthly fish consumption.

		Model 1: Single metal models	Model 2: Fully adjusted	Model 3: Co-adjusted
As	Lowest	0 (ref)	0 (ref)	0 (ref)
	Middle	-3.00 (-7.50, 0.57)	-2.14 (-6.17, 1.89)	-1.97 (-5.86, 1.92)
	Highest	**-4.71 (-9.45, -0.70)**	-4.67 (-9.36, 0.02)	**-4.24 (-8.41, -0.06)**
Ni	Lowest	0 (ref)	0 (ref)	0 (ref)
	Middle	**-8.01 (-11.9, -4.13)**	**-7.10 (-10.9, -3.30)**	**-6.95 (-10.8, -3.15)**
	Highest	-3.66 (-7.63, 0.31)	-2.79 (-6.61, 1.04)	-2.57 (6.39, 1.24)
Cd	Continuous	0.79 (-0.86, 2.43)	1.41 (-0.45, 3.27)	
Hg	Continuous	0.32 (-1.44, 2.09)	0.54 (-1.21, 2.29)	
Pb	Continuous	-0.03 (-1.63, 1.70)	0.21 (-1.72, 2.14)	
U	Continuous	-0.74 (-2.61, 1.12)	-0.80 (-2.94, 1.34)	

TABLE S7. ORDINAL regressions with monthly fish consumption added as a covariate. Models adjusted for sex, age, residential municipality, occupational risk category, and monthly fish consumption.

		Model 1: Single metal models	Model 2: Fully adjusted	Model 3: Co-adjusted
As	Lowest	1 (ref)	1 (ref)	01 (ref)
	Middle	1.20 (0.59, 2.43)	1.06 (0.50, 2.24)	1.01 (0.49, 2.09)
	Highest	**2.16 (1.06, 4.51)**	2.26 (0.99, 5.30)	2.07 (1.00, 4.34)
Ni	Lowest	1 (ref)	1 (ref)	1 (ref)
	Middle	**2.28 (1.18, 4.53)**	**2.53 (1.28, 5.16)**	**2.34 (1.19, 4.70)**
	Highest	1.44 (0.71, 2.96)	1.45 (0.70, 3.05)	1.33 (0.65, 2.73)
Cd	Continuous	0.85 (0.62, 1.13)	0.71 (0.47, 1.02)	
Hg	Continuous	0.96 (0.71, 1.29)	0.98 (0.71, 1.34)	
Pb	Continuous	0.96 (0.73, 1.26)	0.91 (0.63, 1.29)	
U	Continuous	1.23 (0.90, 1.71)	1.32 (0.90, 1.93)	

TABLE S8. LINEAR regressions restricted to non-smokers (n=245), i.e. excluding smokers. Models adjusted for sex, age, residential municipality, and occupational risk category.

		Model 1: Single metal models	Model 2: Fully adjusted	Model 3: Co-adjusted
As	Lowest	0 (ref)	0 (ref)	0 (ref)
	Middle	**-4.49 (-8.83, -0.14)**	-3.23 (-7.74, 1.27)	-3.30 (-7.59, 0.99)
	Highest	-4.11 (-8.77, 0.56)	-3.63 (-8.92, 1.66)	-3.41 (-8.01, 1.20)
Ni	Lowest	0 (ref)	0 (ref)	0 (ref)
	Middle	**-8.37 (-12.5, -4.20)**	**-8.01 (-12.2, -3.79)**	**-7.88 (-12.1, -3.67)**
	Highest	**-4.28 (-8.53, -0.03)**	-3.89 (-8.20, 0.42)	-3.85 (-8.15, 0.44)
Cd	Continuous	0.93 (-0.85, 2.72)	1.64 (-0.38, 3.66)	
Hg	Continuous	0.19 (-1.60, 1.98)	0.61 (-1.18, 2.39)	
Pb	Continuous	-0.09 (-2.01, 1.83)	-0.20 (-2.47, 2.07)	
U	Continuous	-1.29 (-4.35, 1.76)	-1.39 (-4.94, 2.15)	

TABLE S9. ORDINAL regressions restricted to non-smokers (n=245), i.e. excluding smokers. Models adjusted for sex, age, residential municipality, and occupational risk category.

		Model 1: Single metal models	Model 2: Fully adjusted	Model 3: Co-adjusted
As	Lowest	1 (ref)	1 (ref)	1 (ref)
	Middle	1.41 (0.65, 3.06)	1.14 (0.49, 2.64)	1.14 (0.51, 2.52)
	Highest	1.78 (0.79, 4.05)	1.74 (0.66, 4.60)	1.60 (0.70, 3.69)
Ni	Lowest	1 (ref)	1 (ref)	1 (ref)
	Middle	**3.00 (1.38, 6.95)**	**3.13 (1.39, 7.49)**	**2.97 (1.34, 6.97)**
	Highest	2.22 (0.98, 5.26)	2.19 (0.93, 5.39)	2.03 (0.89, 4.89)
Cd	Continuous	0.81 (0.57, 1.12)	0.67 (0.42, 1.02)	
Hg	Continuous	0.97 (0.71, 1.31)	0.93 (0.66, 1.30)	
Pb	Continuous	0.93 (0.67, 1.28)	0.94 (0.60, 1.45)	
U	Continuous	1.34 (0.79, 2.30)	1.60 (0.85, 3.04)	

TABLE S10. LINEAR regressions with previous year's eGFR added as a covariate. Models adjusted for sex, age, residential municipality, occupational risk category, and 2022 eGFR.

		Model 1: Single metal models	Model 2: Fully adjusted	Model 3: Co-adjusted
As	Lowest	0 (ref)	0 (ref)	0 (ref)
	Middle	-2.34 (-5.77, 1.08)	-1.97 (-5.52, 1.58)	-1.38 (-4.80, 2.04)
	Highest	-2.33 (-6.33, 1.12)	-3.09 (-7.21, 1.03)	-1.96 (-5.63, 1.71)
Ni	Lowest	0 (ref)	0 (ref)	0 (ref)
	Middle	**-5.48 (-8.78, -2.17)**	**-5.07 (-8.45, -1.69)**	**-5.25 (-8.601, -1.89)**
	Highest	-1.52 (-4.91, 1.86)	-1.19 (-4.62, 2.25)	-1.31 (-4.73, 2.10)
Cd	Continuous	0.44 (-0.97, 1.86)	0.46 (-1.13, 2.13)	
Hg	Continuous	0.32 (-1.11, 1.76)	0.43 (-0.96, 1.94)	
Pb	Continuous	0.01 (-1.42, 1.43)	-0.13 (-1.94, 1.45)	
U	Continuous	0.76 (-0.86, 2.39)	0.94 (-0.90, 2.88)	

TABLE S11. ORDINAL regressions with previous year's eGFR added as a covariate. Models adjusted for sex, age, residential municipality, occupational risk category, and 2022 eGFR.

		Model 1: Single metal models	Model 2: Fully adjusted	Model 3: Co-adjusted
As	Lowest	1 (ref)	1 (ref)	1 (ref)
	Middle	1.08 (0.49, 2.37)	0.99 (0.44, 2.27)	0.92 (0.41, 2.06)
	Highest	1.70 (0.76, 3.84)	1.89 (0.75, 4.81)	1.79 (0.70, 3.66)
Ni	Lowest	1 (ref)	1 (ref)	1 (ref)
	Middle	**2.51 (1.18, 5.57)**	**2.60 (1.19, 4.81)**	**2.54 (1.18, 5.74)**
	Highest	1.50 (0.67, 3.48)	1.47 (0.64, 3.45)	1.43 (0.63, 3.33)
Cd	Continuous	0.87 (0.61, 1.21)	0.82 (0.54, 1.23)	
Hg	Continuous	0.90 (0.66, 1.24)	0.90 (0.64, 1.26)	
Pb	Continuous	0.93 (0.68, 1.26)	0.90 (0.61, 1.31)	
U	Continuous	1.02 (0.71, 1.48)	1.06 (0.70, 1.62)	

FIGURE S3: Trace plots from BKMRhat and coda packages between the assessment of continuous eGFR and the primary metal mixture (As, Cd, Hg, Ni, Pb, U).

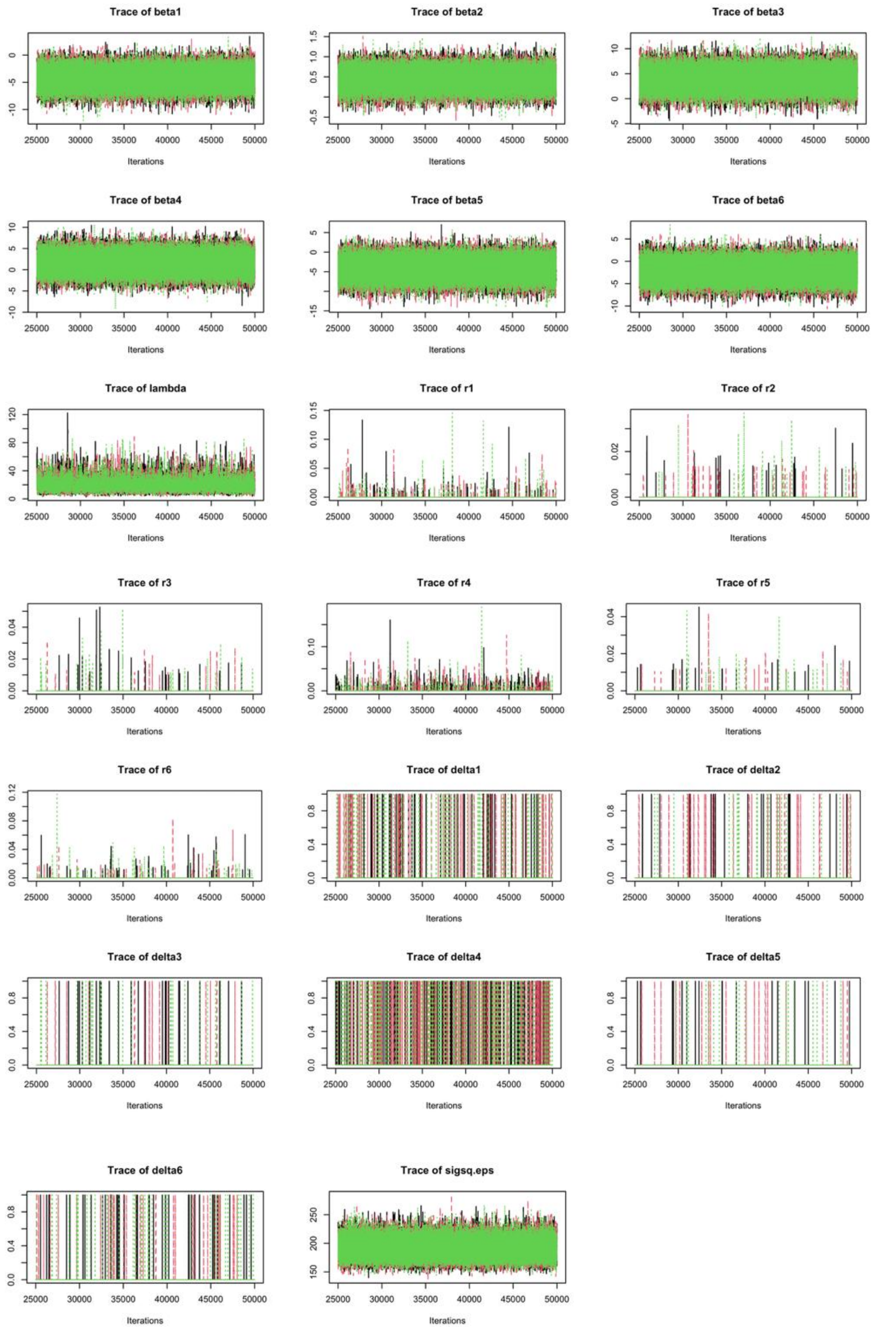

FIGURE S4: BKMR Autocorrelation plot from the combined kmfit for the primary metal set

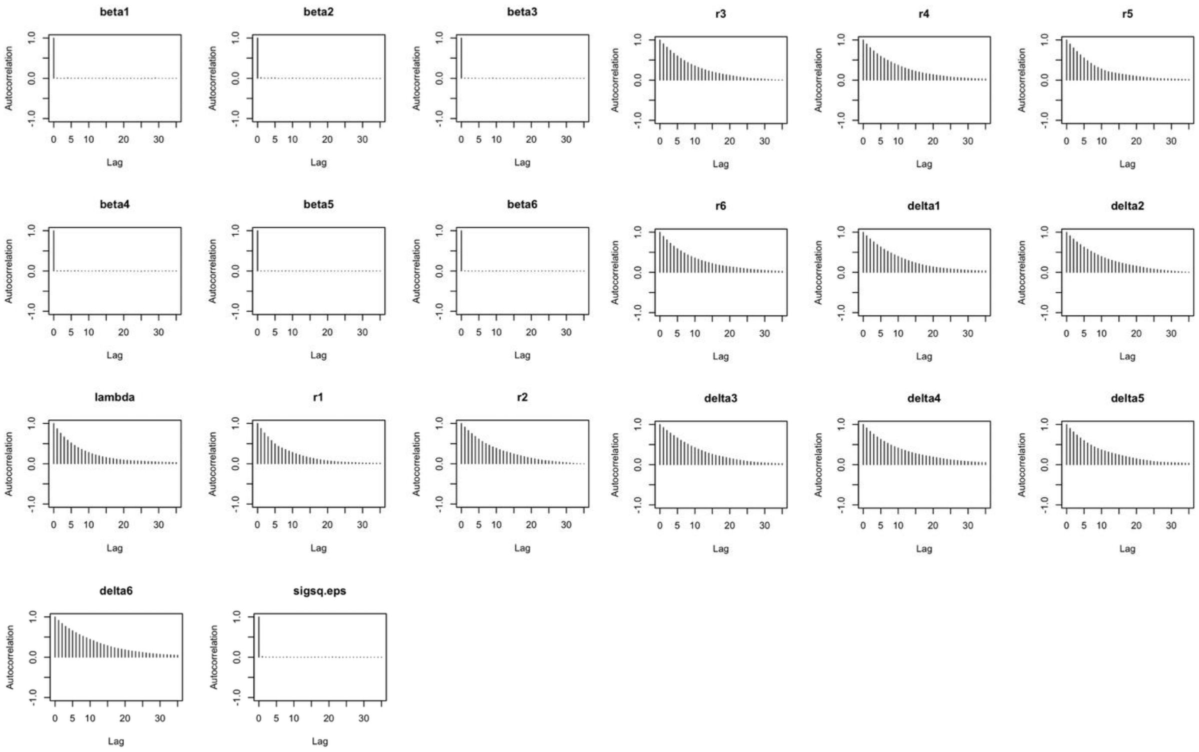

FIGURE S5: BKMR Cross correlation plot from the combined kmfit for the primary metal set (after combined all 3 chains)

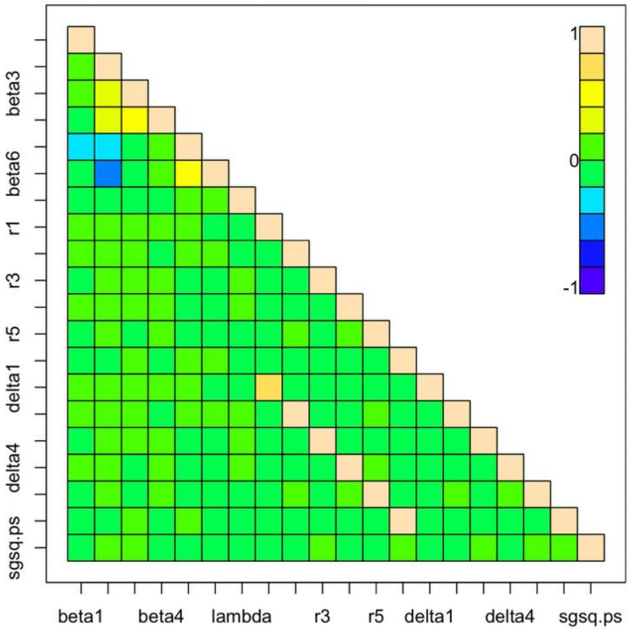

TABLE S12: Diagnostics of BKMR ran with 3 chains and 50k iterations/chain on the PRIMARY metal set.

	Gelman's rhat (upper CI)	Effective sample size
Beta1 (male)	1.00 (1.00)	71326.7
Beta2 (age)	1.00 (1.00)	69356.9
Beta3 (Municipality_LaPazCentro)	1.00 (1.00)	71534.8
Beta4 (Municipality_MinaLimon)	1.00 (1.00)	69446.1
Beta5 (high risk work category)	1.00 (1.00)	75000.0
Beta6 (low risk work category)	1.00 (1.00)	74431.7
lambda	1.00 (1.00)	4859.9
r1- As	1.00 (1.00)	5056.3
r2- Cd	1.02 (1.02)	3919.2
r3-Hg	1.02 (1.02)	3979.9
r4-Ni	1.00 (1.00)	3764.3
r5-Pb	1.02 (1.02)	4511.7
r6-U	1.01 (1.01)	4034.9
delta1	1.00 (1.00)	3590.8
delta2	1.03 (1.04)	3888.4
delta3	1.01 (1.01)	3390.5
delta4	1.00 (1.00)	3281.3
delta5	1.01 (1.01)	3644.5
delta6	1.01 (1.01)	3456.8
Sigsq.esp	1.00 (1.00)	69782.4

TABLE S13: Posterior inclusion probabilities (PIPs) from BKMR of the primary metal set. Model adjusted for age, sex, residential municipality, and occupational risk category.

Metal	Posterior Inclusion Probability
**Ni**	0.07
**As**	0.03
**U**	0.02
**Cd**	0.01
**Hg**	0.01
**Pb**	0.01

TABLE S14: Effect estimates from BKMR of the overall metal mixture (lnAs, lnCd, lnHg, lnNi, lnPb, lnU) with eGFR when all components are held at different percentiles, compared to when all components are at their 50th percentiles. Model adjusted for age, sex, residential municipality, and occupational risk category.

Percentile	Estimate (95% Credible Interval)
**10th**	0.66 (-2.54, 3.86)
**20th**	0.47 (-1.55, 2.50)
**30th**	0.32 (-0.95, 1.59)
**40th**	0.16 (-0.42, 0.74)
**50th**	0.00 (ref)
**60th**	-0.26 (-0.98, 0.45)
**70th**	-0.57 (-2.09, 0.95)
**80th**	-0.91 (-3.28, 1.45)
**90th**	-1.45 (-5.13, 2.23)

=

FIGURE S6: Trace plots from BKMRhat between the assessment of continuous eGFR and the full element mixture, n=20 (Ag, Al, As, Ba, Cd, Co, Cr, Cu, Fe, Hg, Mn, Mo, Ni, Pb, Sb, Se, Sr, Tl, U, Zn).

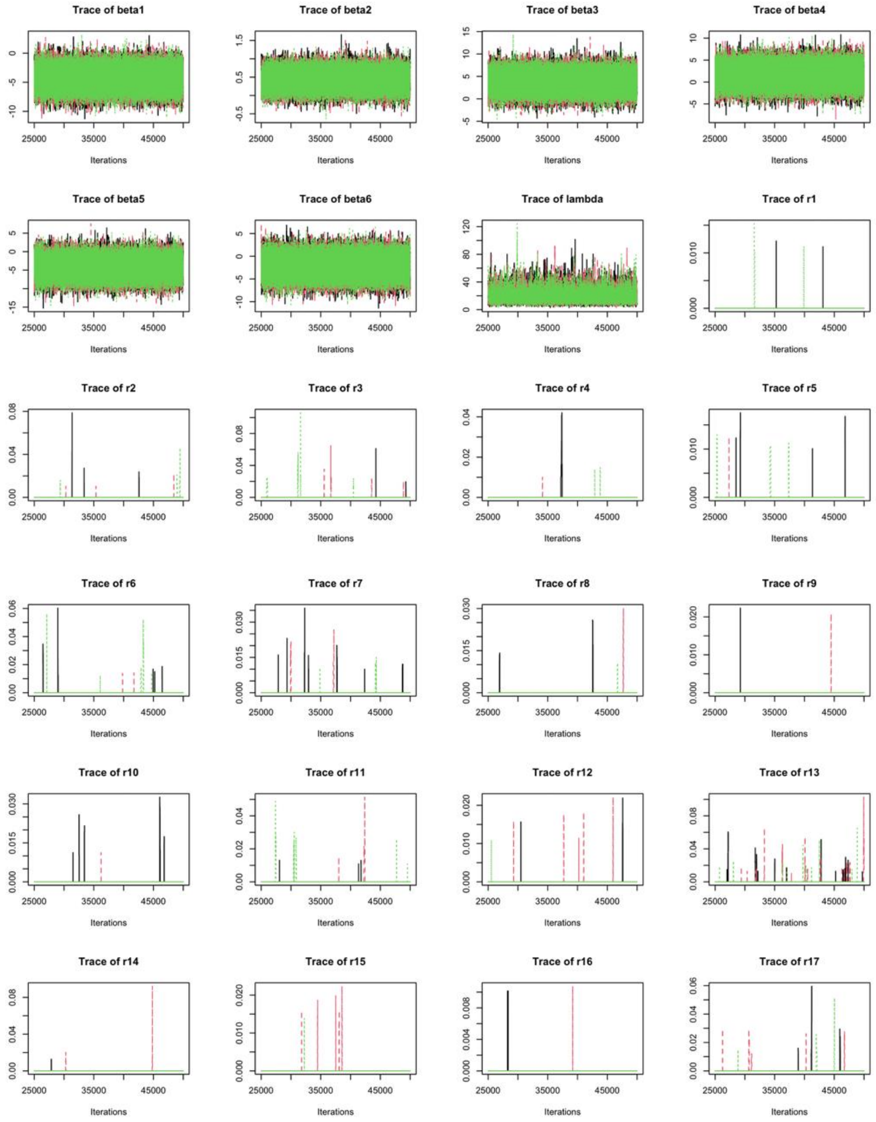

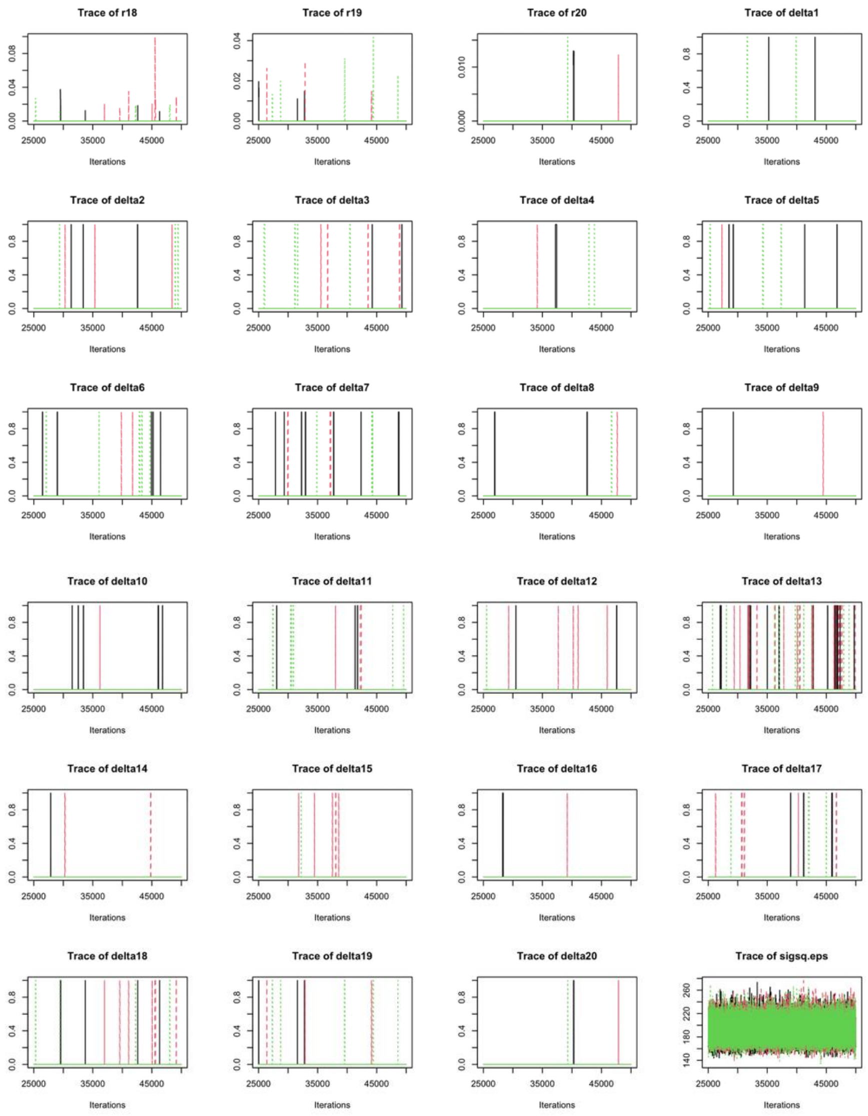
Trace of r18
Trace of r19
Trace of r20
Trace of delta1
Trace of delta2
Trace of delta3
Trace of delta4
Trace of delta5
Trace of delta6
Trace of delta7
Trace of delta8
Trace of delta9
Trace of delta10
Trace of delta11
Trace of delta12
Trace of delta13
Trace of delta14
Trace of delta15
Trace of delta16
Trace of delta17
Trace of delta18
Trace of delta19
Trace of delta20
Trace of sigsq.eps
Iterations
25000
35000
45000

FIGURE S7: BKMR Autocorrelation plots from BKMRhat between the assessment of continuous eGFR and the full element mixture, n=20 (Ag, Al, As, Ba, Cd, Co, Cr, Cu, Fe, Hg, Mn, Mo, Ni, Pb, Sb, Se , Sr, Tl, U, Zn).

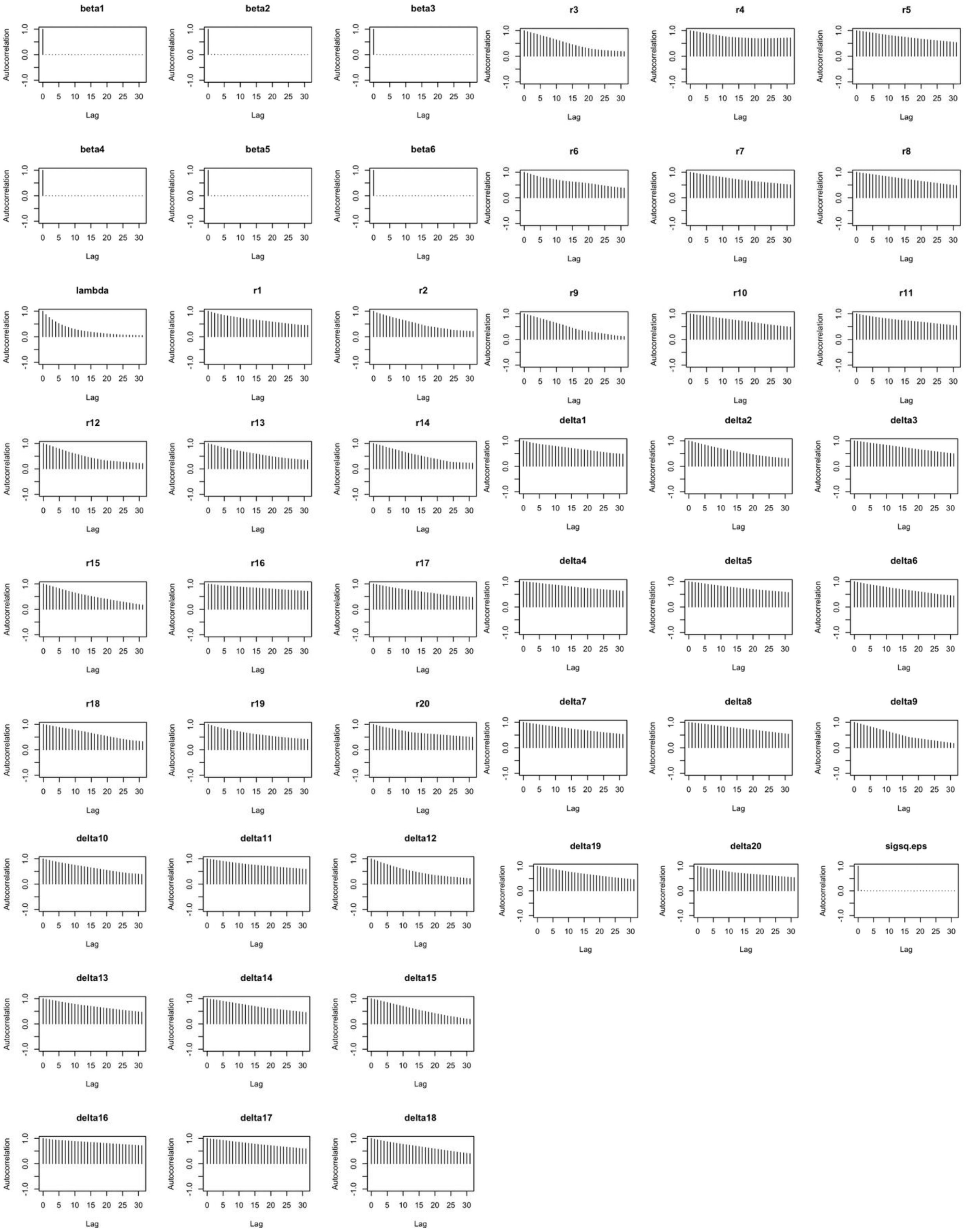

FIGURE S8: BKMR Crosscorrelation plots from BKMRhat between the assessment of continuous eGFR and the full element mixture, n=20 (Ag, Al, As, Ba, Cd, Co, Cr, Cu, Fe, Hg, Mn, Mo, Ni, Pb, Sb, Se, Sr, Tl, U, Zn).

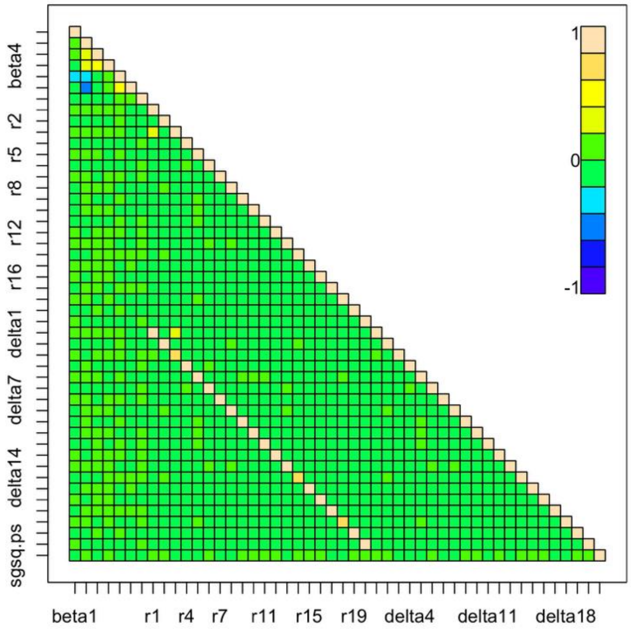

TABLE S15: Diagnostics of BKMRhat between the assessment of continuous eGFR and the full element mixture, n=20 (Ag, Al, As, Ba, Cd, Co, Cr, Cu, Fe, Hg, Mn, Mo, Ni, Pb, Sb, Se, Sr, Tl, U, Zn).

	Gelman's rhat	Effective sample size
Beta1 (male)	1.00 (1.00)	73838.3
Beta2 (age)	1.00 (1.00)	70304.2
Beta4 (Municipality_LaPazCentro)	1.00 (1.00)	74352.8
Beta5 (Municipality_MinaLimon)	1.00 (1.00)	74503.4
Beta8 (high risk work category)	1.00 (1.00)	74236.3
Beta9 (low risk work category)	1.00 (1.00)	70041.0
lambda	1.00 (1.00)	4492.7
r1- Ag	1.26 (1.34)	6531.6
r2- Al	1.08 (1.08)	3405.9
r3-As	1.15 (1.17)	2355.0
r4-Ba	1.21 (1.25)	1366.3
r5-Cd	1.12 (1.14)	3709.2
r6-Co	1.10 (1.12)	1813.7
R7-Cr	1.07 (1.08)	1542.8
R8-Cu	1.06 (1.07)	1156.3
R9-Fe	1.12 (1.13)	1871.4
R10-Hg	1.29 (1.49)	8427.4
R11-Mn	1.11 (1.13)	1552.7
R12-Mo	1.10 (1.11)	7986.3
R13-Ni	1.02 (1.02)	1335.7
R14-Pb	1.27 (1.33)	1756.8
R15-Sb	1.23 (1.28)	1518.7
R16-Se	1.27 (1.38)	6493.5
R17-Sr	1.02 (1.02)	812.8
R18-Tl	1.14 (1.15)	1585.7
R19-U	1.22 (1.14)	1793.1
R20-Zn	1.18 (1.20)	10291.1
Sigsq.esp	1.00 (1.00)	68999.4

FIGURE S9: Results (univariate relationships and overall mixture effect) from BKMR ran between the assessment of continuous eGFR and the full element mixture, n=20 (Ag, Al, As, Ba, Cd, Co, Cr, Cu, Fe, Hg, Mn, Mo, Ni, Pb, Sb, Se, Sr, Tl, U, Zn).

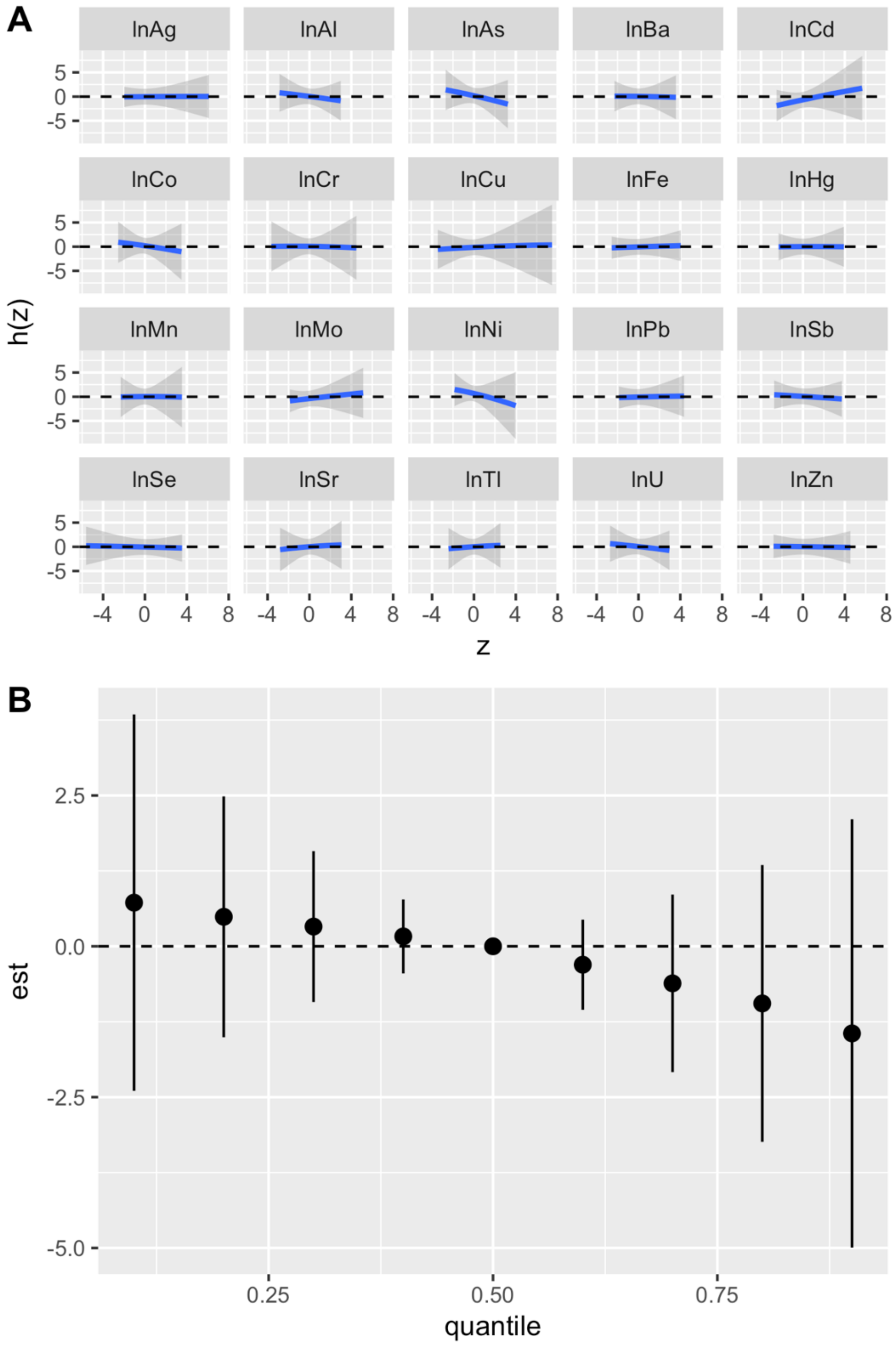

TABLE S16. PIPs from BKMR with complete element set (n=20)

Element	Posterior Inclusion Probability
Ag	0.002
Al	0.004
As	0.008
Ba	0.004
Cd	0.006
Co	0.006
Cr	0.008
Cu	0.004
Fe	0.001
Hg	0.003
Mn	0.007
Mo	0.002
**Ni**	**0.023**
Pb	0.002
Sb	0.003
Se	0.002
Sn	0.010
Sr	0.007
Tl	0.001
U	0.006
Zn	0.001

SUPPLEMENTARY DISCUSSION: Metal biomarkers and applications to forward and reverse causality

As outlined in the main Discussion section, our use of toenail biomarkers has allowed for further exploration of the temporal relationship between metal exposure and kidney function, given that toenails reflect exposures occurring several months before the collection of serum creatinine for GFR estimation.

In this Supplement, we will focus on the relationship between toenail Ni and eGFR, as this relationship was the most consistent and showed the strongest effect across all main and sensitivity regression models. The following text will describe two exposure scenarios—(1) assuming forward causation in accordance with our working hypothesis, that exposure to select heavy metals causes subsequent kidney damage that could be detectable through decreased eGFR; and (2) reverse causation, in that our main outcome of interest, kidney function, is actually responsible for the observed toenail biomarker concentrations of Ni exposure. This concept is discussed thoroughly, though in regard to urine and serum biomarkers of exposure, by Weaver et al. in their 2016 work titled "Challenges for environmental epidemiology research: are biomarker concentrations altered by kidney function or urine concentration adjustment?" (DOI 10.1038/jes.2015.8).

The following assessment is simplified and built on many assumptions, including that the distribution and metabolism of absorbed Ni occur at a constant rate for all levels of both Ni exposure and kidney function. While this is likely a biologically implausible assumption, we will employ the assumption to make further queries on the forwardly or reversely causal relationship between toenail Ni and eGFR as to be understood by our data. Of note, reverse causation may not be as much of a concern in healthy kidney states, with some nephrologists suggesting that it is only of concern for biomonitoring studies when eGFR<60 ml/min/1.73m^2.

Under these assumptions, we could explore the following two scenarios, which are displayed in the below graphs:

(1) Forward causation: that exposure to heavy metals causes lowered eGFR

(2) Reverse causation: that lower eGFR (decreased kidney filtration) causes decreased urinary excretion of Ni (as supported by Jin et al., 2018, DOI 10.1016/j.envint.2018.11.002), which causes increased serum Ni. Increased serum Ni would allow for greater deposition into toenails. Under this framework, *lower eGFR could explain higher toenail Ni levels, and higher eGFR could explain lower toenail Ni levels.*

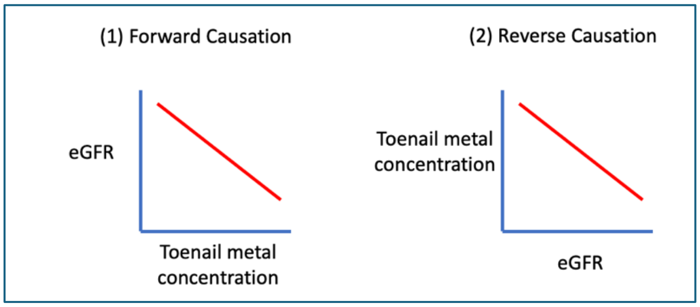

Through graphs (1) and (2), we see that a negative relationship between toenail Ni and eGFR may be explained by forward or reverse causation, hypothetically.

It is important for us to consider another scenario in which increased metal exposure causes a different type of kidney function change, such as hyperfiltration. Hyperfiltration reflects a scenario where kidney injury has occurred, and the renal system reacts by entering a hyper-active state of increased filtration often preceding a sharp decrease in kidney function. The following graph displays the expected relationship in a forwardly causal-hyperfiltration scenario:

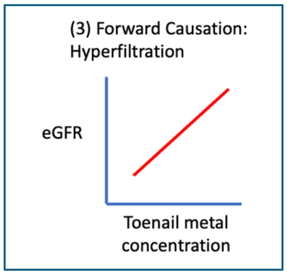

If a trend like in graph (3) were observed, it would be difficult to explain as related to reverse causation, if the assumption of decreased kidney function as causing decreased urinary excretion were to hold true at all levels of exposure and eGFR. Another possible, though unlikely, explanation for a trend such as this would be that exposure to nephrotoxic metals is beneficial for kidney health, though that concept is strongly contradictory to the existing body of animal- and cell-based toxicological studies (especially at Ni exposure levels observed in our cohort, which appear to be highly elevated compared to global cohorts). Therefore, an observation of such may imply a forwardly causal relationship between metal exposure and the outcome of hyperfiltration (or temporarily elevated eGFR).

Therefore, when assessing the U-shaped response curve of toenail Ni on eGFR as observed in our data, we can understand different aspects of this curve as representing different scenarios (results below from a fully adjusted generalized additive model with a restricted cubic spline and 10 knots; pulled from Figure 2).

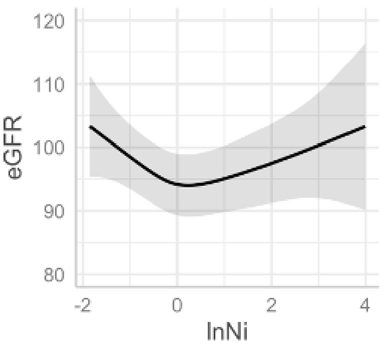

The left-hand side of the graph shows a negative relationship between Ni and eGFR. From the above discussion, this trend could be a candidate for either forward causation (that increased Ni exposure caused decreased eGFR) or reverse causation (that decreased eGFR caused increased Ni toenail concentrations). The right-hand side of the graph, however, shows a positive relationship between Ni and eGFR. This trend could likely be a candidate for forwardly causal hyperfiltration, in that increased Ni exposure (evidenced through increased Ni toenail concentrations) caused an increase in eGFR. Here, these hypothesized conclusions describe visual trends and do not rely on statistical significance. A caveat that this non-linear dose response could also be partially explained by random variability in our relatively small sample, as said in the main text.

We further explored the possibility of hyperfiltration in our dataset. The highest eGFR measurement was 126 ml/min/1.73m^2, which is lower than common hyperfiltration thresholds (>140 or 160 ml/min/1,73m^2). When we select the participants with eGFR above the 95th percentile (>114 ml/min/1.73m^2), those 15 individuals have a median Ni concentration of 5.21 ug/g (Q1, Q3: 3.59, 10.0), which is more than 50% lower than the median of the general population at 11.7 ug/g (Q1, Q3: 6.53, 28.26), demonstrating that these individuals are likely on the left-hand side of the above eGFR~Ni curve.

From a different perspective, we also assessed those with the highest Ni exposure. The 15 participants with toenail Ni in the 95th percentile or higher (>132 ug/g) had a median eGFR of 109.7 ml/min/1.73m^2 (IQR 9.98). This is 7 units higher than the eGFR of the total study population (102.3 ml/min/1.73m^2; IQR 20.0). In the year prior to when toenails were collected, the same group of individuals with extremely high Ni exposure had median eGFR of 106.1 units (IQR 17.3). This median eGFR increase of 3 ml/min/1.73m^2 across one year, during which Ni exposure did occur, may provide evidence of hyperfiltration developing due to extremely high Ni exposure (both relative to our population and in comparison to external toenail datasets).

While this exploration is strengthened by our use of a historical biomarker of exposure, we are still limited by the fact that this is a single measurement of both the exposure and outcome of interest. We have descriptively explored eGFR data from the year previous to when toenails were collected, though the vast uncertainty surrounding timing of metal deposition into toenails (and differences of those rates across both metals and (likely) participant ages and toenail growth rates, let alone baseline kidney

function) seems an important barrier past which we should not force further assumptions.

Therefore, this work would be strongly supported by studies with repeated longitudinal collection of both toenails and serum creatinine or cystatin C for GFR estimation, which would provide an excellent avenue through which to catalogue changes in eGFR with a more correct understanding of exposure timing and relationship temporality. We felt there were too many unsteady assumptions required to assess change in kidney function in relation to a single time point of exposure and thus did not present those results here.
